# Determining hemispheric language dominance from MEG beta-power modulations: Concordance with fMRI

**DOI:** 10.1016/j.neuroimage.2026.122051

**Published:** 2026-06-11

**Authors:** Vahab Youssofzadeh, Jeffrey R. Binder, Joseph Heffernan, Elizabeth Bock, Amit Jaiswal, Jeffrey Stout, Candida Ustine, Rupesh Kumar Chikara, Priyanka Shah-Basak, Colin Humphries, Jed Mathis, Lisa L. Conant, William L. Gross, Chad Carlson, Christopher T. Anderson, Bruce Hermann, Beth Meyerand, Manoj Raghavan

**Affiliations:** a Neurology, Medical College of Wisconsin, Milwaukee, WI, USA; b Biophysics, Medical College of Wisconsin, Milwaukee, WI, USA; c MEGIN Oy, Espoo, Finland; d Department of Neuroscience and Biomedical Engineering, Aalto University School of Science, Espoo, Finland; e Anesthesiology, Medical College of Wisconsin, Milwaukee, WI, USA; f Neurology, University of Wisconsin-Madison, Madison, WI, USA; g Radiology, University of Wisconsin-Madison, Madison, WI, USA; h Medical Physics, University of Wisconsin-Madison, Madison, WI, USA; i Biomedical Engineering, University of Wisconsin-Madison, Madison, WI, USA

**Keywords:** Magnetoencephalography (MEG), Beta band power suppression, Language lateralization, Temporal lobe epilepsy (TLE), Laterality index, MEG–fMRI concordance, Participant/ROI-specific timing

## Abstract

Accurate identification of the language-dominant hemisphere is essential for surgical planning in drug-resistant focal epilepsy. Functional MRI is widely used as a noninvasive alternative to the Wada test but may be contraindicated or unreliable in some patients, motivating complementary approaches such as magnetoencephalography (MEG). We tested whether beta-band MEG power suppression can provide reliable language lateralization when combined with a task-matched nonlinguistic control, participant/ROI-specific timing, and a multi-threshold weighted-bootstrap laterality index (LI). Seventy-four adults with temporal lobe epilepsy completed visual semantic-decision and false-font control tasks during MEG. Beta-band (17–25 Hz) source power was estimated with DICS beamforming, and LIs were computed in frontal, temporal, angular, and composite lateral ROIs using 300-ms sliding windows. For comparison, participants also completed an auditory semantic-decision versus tone-decision fMRI protocol, with fMRI LIs derived from matched ROIs. MEG showed a left-lateralized spatiotemporal sequence in which frontal LI emerged earlier than temporal–parietal LI, following early bilateral temporo-parieto-occipital engagement. Compared with a prestimulus baseline, the false-font control contrast increased LI magnitude and improved MEG–fMRI correspondence, with a peak MEG–fMRI correlation of *r* = 0.65. Participant/ROI-specific timing produced only small and mixed changes in concordance. Using the optimized MEG pipeline, MEG and fMRI agreed in 67/74 patients (90.5%; 95% CI: 83–96%; κ = 0.81) Bootstrap LIs performed similarly to magnitude- and vertex-count-based variants while providing confidence intervals. These findings suggest that beta-band MEG suppression during semantic decision, especially when contrasted with a nonlinguistic control task, provides a temporally resolved and clinically feasible marker of hemispheric language dominance that is highly concordant with fMRI.

## 1. Introduction

Resective or ablative surgery can be an effective treatment for a large proportion of patients with drug-resistant focal epilepsy (de Tisi et al., 2011; Wiebe et al., 2001). However, postoperative neurocognitive deficits, especially in language and memory, are a significant concern when planning surgery in the dominant hemisphere (Helmstaedter, 2004; Helmstaedter and Kurthen, 2001; Hermann et al., 1992). Identification of the language-dominant hemisphere has therefore been a key element of presurgical evaluations for many decades. Despite this, approximately 30–50% of patients undergoing dominant (left) temporal lobe resection show postoperative naming decline, and approximately 17–25% exhibit substantial or persistent word-finding difficulties that can affect quality of life (Busch et al., 2018; Hamberger, 2015; Langfitt and Rausch, 1996). Quantitative estimates of language dominance established using functional MRI (fMRI) combined with neuropsychological variables have been shown to predict naming outcome (Bonelli et al., 2012; Gross et al., 2022; Sabsevitz et al., 2003; Trimmel et al., 2019) and verbal memory decline (Binder et al., 2008a; Gross et al., 2025), underscoring the clinical value of accurate language lateralization assessments.

The intracarotid amobarbital procedure (IAP; Wada test) has long served as the reference for hemispheric language dominance, but it is invasive and carries a nontrivial complication rate (approximately 3%; Loddenkemper et al., 2008). fMRI is the most widely adopted noninvasive alternative, although MRI may be contraindicated in some individuals due to the strong magnetic fields involved. Because it is based on blood flow changes, fMRI may also give invalid results in the presence of neurovascular uncoupling (Pillai and Mikulis, 2015). Its relatively low temporal resolution also makes fMRI highly susceptible to signal degradation related to head movements, which may present a particular problem in pediatric cohorts and cognitively impaired individuals. Beyond task-fMRI and the IAP, centers also use intraoperative or extraoperative cortical stimulation and intracranial EEG primarily for language localization, which can indirectly inform hemispheric dominance when mapping yields strongly lateralized essential sites. Other methods explored for dominance include navigated transcranial magnetic stimulation (nTMS) (Jeltema et al., 2021; Lehtinen et al., 2018; Picht et al., 2013), resting-state fMRI when task compliance is limited (Chiang et al., 2017; Pur et al., 2021; Rosazza et al., 2018), and, in select contexts, functional near-infrared spectroscopy (fNIRS) and behavioral measures such as dichotic listening (Arun et al., 2018; Fernandes et al., 2006). While the feasibility of determining hemispheric language dominance using these latter methods has been demonstrated in small studies, they have yet to be validated at scale. Concordance with invasive benchmarks varies across modalities and cohorts, and method choice is often constrained by patient tolerance and local expertise (Dym et al., 2011; Janecek et al., 2013a).

Magnetoencephalography (MEG) has been endorsed in clinical guidelines as a noninvasive adjunct for presurgical mapping prior to neurosurgical procedures (Bagić et al., 2011; Baillet, 2017; Burgess et al., 2011; Papanicolaou et al., 2004; Stufflebeam, 2011). Lateralization of language using MEG has been demonstrated using two types of cortical responses: evoked responses (phase-locked event-related fields, ERFs) and oscillatory power changes (Doss et al., 2009; Hirata et al., 2004; Papanicolaou et al., 2004; Raghavan et al., 2017; Salmelin, 2007; Tanaka et al., 2009; Youssofzadeh et al., 2020). Evoked response source imaging using equivalent current dipoles or noise-normalized minimum norm solutions (dSPM; Dale et al., 2000) has shown high concordance with the Wada test for estimating language dominance in several studies. For example, auditory ERFs during a word recognition task analyzed using single dipole modeling methods found an 85% concordance with the IAP for ternary classification of dominance into left, right, or symmetric laterality (Papanicolaou et al., 2004), while dSPM mapping of ERFs produced by a visual semantic-decision achieved about 91% agreement with IAP in 35 patients (Tanaka et al., 2013). A parallel approach to language dominance estimation using MEG is based on task-related beta band (~ 15–25 Hz) power suppression (event-related desynchronization, or ERD), which tolerates trial-to-trial latency jitter and often yields stable lateralization with fewer trials, compared to ERF protocols (Findlay et al., 2012; Herfurth et al., 2022; Hinkley et al., 2020; Pang et al., 2011; Wang et al., 2012; Youssofzadeh et al., 2022, 2020). Critically, beta suppression correlates with local multi-unit firing and BOLD increases (Kilavik et al., 2013; Logothetis et al., 2001; Singh et al., 2002). Using beta suppression, a silent reading paradigm reproduced IAP dominance in 19 of 20 surgical candidates (Hirata et al., 2004), and neuromagnetic oscillatory mapping aligned closely with invasive procedures for both dominance and localization (Hirata et al., 2010). In a pediatric cohort, beta band MEG during verb generation also showed strong concordance with fMRI (Pang et al., 2011), and in adults, beta power decrease mapping demonstrated robust inferior frontal laterality with regionally variable MEG–fMRI overlap and IAP validation (Herfurth et al., 2022).

Despite these successes, reported MEG concordance with IAP or fMRI still spans approximately 70–95% for ternary classification of language dominance (Trébuchon et al., 2020). In this study, we evaluated three methodological variables that may contribute to this spread. The first of these is the selection of an optimal baseline state against which the language task is contrasted. In fMRI studies, replacing a “resting” baseline with a modality-matched nonlinguistic control task (e.g., perceptual decisions on false fonts, tones, or scrambled pictures) reduces bilateral sensorimotor and domain-general attention contributions to the activation map and yields stronger, more left-lateralized language responses (Binder et al., 2008b; Findlay et al., 2012; Seghier, 2008; Wang et al., 2012). MEG studies, however, typically employ a prestimulus resting period as a local baseline, which provides no controls for these nonlinguistic factors. Second is the selection of an optimal temporal window: although many ERF/dSPM language-mapping studies compute LIs in canonical mid-latency bins (often 250–600 ms), inter-individual and regional latency differences can shift the true peak outside any single bin (Kochi et al., 2023; McDonald et al., 2009; Raghavan et al., 2017; Tanaka et al., 2013). Peak beta suppression also tends to occur approximately 50–100 ms earlier in the inferior frontal than in the superior/middle temporal cortex and varies by >300 ms across individuals. Fixed windows can therefore miss the maximum, whereas peak-aligned windows have improved invasive concordance in prior series (Bourguignon et al., 2013; Tanaka et al., 2013). The third variable is the laterality index (LI) estimation algorithm: thresholded magnitude and vertex-count LIs are sensitive to residual noise and threshold choice, whereas a weighted average, multi-threshold LI generated with bootstrap resampling provides more stable estimates with explicit confidence intervals, especially in heterogeneous clinical cohorts (Findlay et al., 2012; Wilke and Schmithorst, 2006).

Here, we systematically compared three methodological choices in MEG language mapping: (i) contrast type (prestimulus baseline vs. a task-matched nonlinguistic control), (ii) timing strategy (a fixed window vs. per-participant, per-ROI temporal windows guided by a regional response index [rRI]), and (iii) laterality estimator (suprathreshold magnitude summation, suprathreshold vertex counting, or a weighted-bootstrap LI with confidence intervals). We applied these alternatives to beta-band suppression during a visual semantic-decision task contrasted with a nonlinguistic symbol-matching task in 74 adults with TLE, each of whom also underwent 3T fMRI using an extensively validated semantic-decision > tone-decision protocol. We then asked how these methodological choices influence MEG–fMRI concordance and whether their effects are complementary, treating fMRI as a noninvasive reference standard for cross-modal comparison rather than as definitive ground truth for hemispheric language dominance. We also examined whether participant- and ROI-specific factors, recording-quality metrics, and rRI-based measures of response strength help explain discordances between MEG- and fMRI-derived language laterality classifications.

## 2. Material and methods

### 2.1. Participants

We analyzed MEG and fMRI data from 74 adults (mean age ± SD = 39.1 ± 11.9 years; range = 19–60) enrolled in the Epilepsy Connectome Project (ECP; U01NS093650), a joint initiative of the Medical College of Wisconsin and the University of Wisconsin–Madison integrating neuroimaging, electrophysiology, and behavioral phenotyping in individuals with TLE. The full ECP cohort is larger; for the present study, we included only those participants who completed both the MEG and fMRI language-mapping protocols and had complete, analyzable datasets. Of the 81 ECP participants who underwent MEG recording, 74 met these criteria and were included in the current analyses. The remaining 7 were excluded because they did not complete the fMRI language protocol (*n* = 4), were missing the second run of the MEG task (*n* = 1), or did not complete the MEG semantic-decision task (*n* = 2).

All included participants were native English speakers. Handedness, assessed with the Edinburgh Handedness Inventory when available (Oldfield, 1971), was predominantly right-handed: 59/74 right-handed, 9/74 left-handed, 4/74 ambidextrous, and 2/74 missing. Most patients had left TLE: 51/74 left TLE, 15/74 right TLE, 4/74 bilateral TLE, 2/74 uncertain seizure-focus lateralization, and 2/74 missing. Data collection within the ECP and the secondary analyses reported here were approved by the Institutional Review Board of the Medical College of Wisconsin (Milwaukee, WI). All participants provided written informed consent and received an hourly stipend. Demographic and clinical characteristics of the analyzed cohort are summarized in Table 1.

### 2.2. MEG and fMRI tasks

#### 2.2.1. MEG Semantic-decision and symbol-matching tasks

**Fig. 1.**
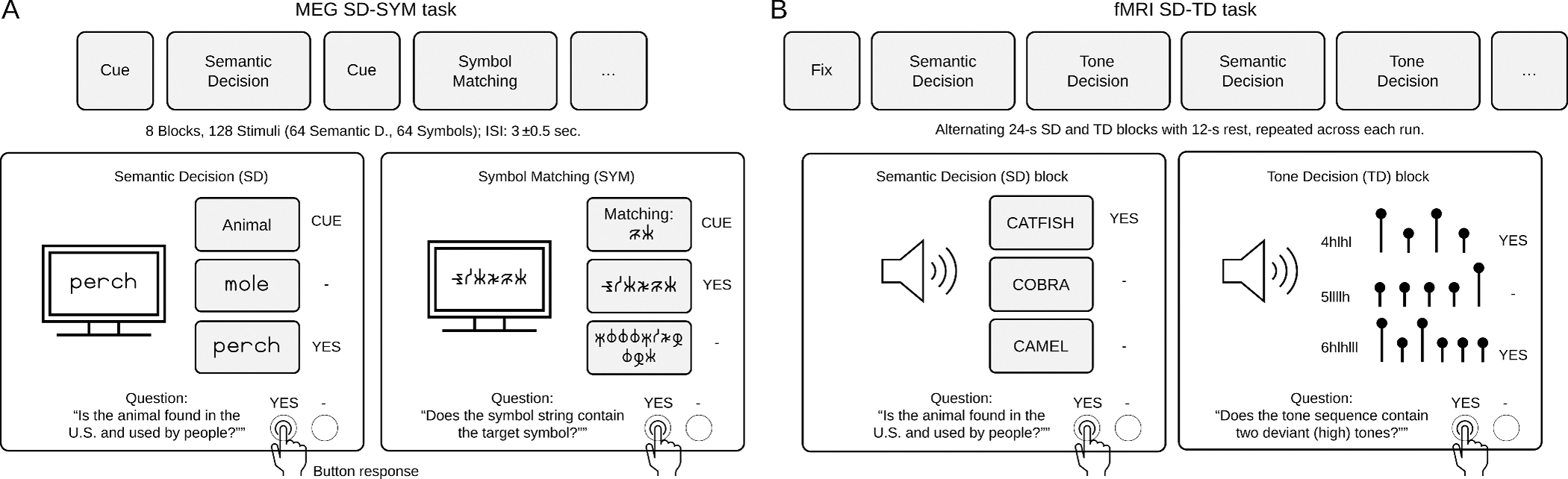
Task schematics for MEG and fMRI language mapping. (A) MEG semantic-decision (SD) and symbol-matching (SYM) task. Each participant completed two runs, each with 16 alternating blocks, 8 SD and 8 SYM, for 256 trials total. In SD blocks, participants viewed animal names and responded only when the animal was both found in the United States and useful to humans. In SYM blocks, participants viewed false-font strings and responded only when a string contained both predefined target glyphs. Each stimulus was presented for 2 s, followed by a 1.0–1.5 s blank interval, yielding a stimulus-onset asynchrony of 3.0–3.5 s. (B) fMRI auditory semantic-decision (SD) versus tone-decision (TD) task. Participants completed two functional runs of approximately 5 min each. Each run contained four 24-s SD blocks and four 24-s TD blocks, interleaved with 12-s rest periods. During SD blocks, animal names were presented every 3 s and participants responded when the animal was both found in the United States and useful to humans. During TD blocks, tone sequences were presented and participants responded when the sequence contained two deviants.

The protocol comprised two blocked conditions: (1) Semantic-decision (SD), in which participants viewed animal names and pressed a button if the animal was both found in the United States and useful to humans (full list in Table S1); and (2) Symbol matching (SYM), in which participants viewed false-font strings and responded only when two predefined target symbols appeared. After standardized instructions and practice, each participant completed two runs (16 blocks per run; 128 trials per run, 64 per condition). Each stimulus was displayed for 2.0 s, followed by a 1.0 s blank plus a random jitter ranging from 0 to 0.5 s; thus, the post-offset interval was 1.0–1.5 s, and the stimulus-onset asynchrony (SOA) was 3.0–3.5 s. Across the two runs, each participant completed 256 MEG trials in total (128 SD, 128 SYM). Given the 3.0–3.5 s SOA, the total task acquisition time was approximately 12.8–15.0 min, excluding instructions and practice. Responses were made on an MEG-compatible response pad. Accuracy and reaction time, measured from stimulus onset, were recorded. A timing diagram is shown in Fig. 1A.

#### 2.2.2. fMRI Semantic-decision vs. tone-decision protocol

We employed an auditory semantic-decision versus tone-decision (SD vs. TD) fMRI protocol validated for language lateralization and for predicting postoperative naming/memory outcomes in TLE (Binder et al., 2008a, 1997; Janecek et al., 2013a). Auditory stimuli were delivered via MR-compatible headphones, and responses were collected with a left-hand MR-safe button box. A schematic of the fMRI task structure and timing is shown in Fig. 1B. The fMRI language-mapping acquisition consisted of two functional runs of approximately 5 min each (10 min total). Each run contained four 24-s SD blocks and four 24-s TD blocks, interleaved with 12-s rest periods. During SD blocks, animal names were presented every 3 s, and participants made a left-hand button press when the animal was both found in the United States and useful to humans. During TD blocks, sequences of standard (low) and deviant (high) tones were presented, and participants made a left-hand button press when the sequence contained two deviants.

Imaging was performed on a 3T GE Discovery MR750 scanner using a 32-channel head array receiver coil. T1-weighted structural images were acquired using a reduced magnetization prepared gradient echo (MPRAGE) sequence with TR/TE = 604 ms/2.516 ms, TI = 1060.0 ms, flip angle = 8°, FOV = 25.6 cm, and voxel size = 0.8 mm isotropic. Functional scans used a whole-brain, simultaneous multi-slice, gradient echo echoplanar sequence (8 bands, 72 slices, TR/TE = 802 ms/33.5 ms, flip angle = 50°, matrix = 104 × 104, FOV = 20.8 cm, voxel size = 2.0 mm isotropic).

### 2.3. MEG Data acquisition and analysis

#### 2.3.1. Data acquisition

MEG was recorded with a 306-channel VectorView^™^ system (Elekta Neuromag, Helsinki, Finland) inside a magnetically shielded room (ETS-Lindgren, Eura, Finland) at Froedtert Hospital, Milwaukee, WI. Signals were sampled at 2 kHz with a 0.03-Hz high-pass filter. Head position was tracked with four HPI coils. Three fiducials (nasion and left/right preauricular points) and >200 scalp points were digitized with a Polhemus FASTRAK (Colchester, VT) for MEG–MRI coregistration. Visual stimuli were delivered using PsychoPy v3.0 (Peirce, 2007) and back-projected (Panasonic DLP PT-D7700U-K) to a screen viewed via a helmet-mounted mirror.

#### 2.3.2. Preprocessing

External interference was suppressed with temporal signal–space separation (tSSS; MaxFilter v2.2, Elekta Neuromag; Taulu and Simola, 2006). Manufacturer cross-talk correction and fine-calibration files were applied. Unless otherwise noted, parameters were Lin=8, Lout=3, 10-s tSSS buffer, subspace correlation limit 0.98, and origin at head center. Continuous HPI data were used for movement compensation (realignment to the median head position per run). Automatic bad-channel detection was enabled and visually confirmed; marked sensors were excluded from the SSS basis and, when necessary, interpolated prior to forward modeling. Post-tSSS outputs were visually inspected; runs were reprocessed if the RMS HPI fit error exceeded 5 mm or if substantial residual artifact was present. Cardiac, ocular, and myogenic artifacts were removed using automated ICA in MEGnet (Treacher et al., 2021) combined with signal-space projection in Brainstorm (Tadel et al., 2011). Data were low-pass filtered at 40 Hz (4th-order Butterworth) and downsampled to 1 kHz. Trials with kurtosis >15 were rejected (mean loss <4%).

#### 2.3.3. DICS Beamformer source modeling

MEG data were coregistered to each participant’s T1 MRI using fiducials and scalp points. Cortical surfaces were reconstructed with FreeSurfer and downsampled to 15,002 vertices. Leadfields were computed with an overlapping-spheres head model (Huang et al., 1999). Beta band (17–25 Hz) source power was estimated with Dynamic Imaging of Coherent Sources (DICS; Gross et al., 2001), following our prior work (Youssofzadeh et al., 2023, 2022, 2020). The 17–25 Hz range was selected a priori to target low-beta event-related desynchronization, guided by prior MEG language-mapping studies and our previous work identifying language-related beta effects in this range (Foley et al., 2019; Herfurth et al., 2022; Kim and Chung, 2008; Youssofzadeh et al., 2020). In the present DICS implementation, cross-spectral density was centered at 21 Hz with ±4-Hz smoothing, yielding effective sensitivity to 17–25 Hz. Sensor-level cross-spectral density (CSD) matrices were computed per epoch using a multitaper FFT (center 21 Hz; ±4-Hz smoothing; 4-s zero-padding) (Mitra and Pesaran, 1999).

A single, condition-independent common spatial filter was derived from pooled epochs (SD + Baseline for SD > Baseline; SD + SYM for SD > SYM) with strong diagonal loading (λ = 100% of mean sensor-space variance) to stabilize the inversion and minimize condition-specific leakage. Dipole orientation at each vertex was fixed to the max-power direction estimated from the common CSD. For condition- and window-specific estimates, the common filter was held fixed and applied to CSDs recomputed separately for each condition/window with minimal additional regularization (λ = 5%). Both gradiometers and magnetometers were used. Source maps were projected to MNI-152 space, smoothed with a 3-mm FWHM Gaussian kernel, and averaged across runs.

At each cortical vertex *v*, we computed the log-power difference (dB) between semantic-decision and control:

$$e(v)=10\left[{log}_{10}P_{SD}(v)-{log}_{10}P_{\text{control}}(v)\right]$$

Here $P_{\text{SD}}$ and $P_{\text{Control}}$ are DICS source-power estimates from SD and the matched control (SYM for SD > SYM; prestimulus for SD > Baseline). Negative values indicate SD-related beta suppression (ERD) relative to control. We then formed a non-negative suppression map s(v)=max{0,−e(v)}, so larger *s* denotes a stronger beta-power decrease.

Group-level inference focused on beta power decreases (SD < control) using a one-sided (negative-tail) cluster-based Monte Carlo permutation test (Maris and Oostenveld, 2007) with 1000 randomizations, cluster-forming t < −5, cluster-mass statistic, and FWE-corrected cluster p < 0.01. For visualization and summary extraction only, cortical maps display |t|(equivalently−tforSD<control) to emphasize cluster extent; all statistical testing used the signed one-sided thresholds described above.

#### 2.3.4. Task contrasts

Beta-band source power (DICS) was estimated in 300-ms sliding windows whose centers ranged from −300 to 1800 ms in 10-ms steps relative to stimulus onset, so that each window covered [t − 150, t + 150] ms around its center t, yielding time-resolved suppression profiles. Source-power estimates were computed separately for each run and then averaged across runs. Thus, each participant contributed up to 128 semantic-decision (SD) trials and 128 symbol-matching (SYM) trials. Using the analyzed interval from −300 to 1800 ms, this corresponds to 268.8 s (~4.5 min) of MEG data per condition contributing to the source-power estimates before trial rejection. Given mean trial loss <4%, the analyzed duration was approximately 4.3 min per condition on average after trial rejection.

Prestimulus baseline definition (for SD > Baseline). The baseline was defined as the −300 to 0 ms interval preceding stimulus onset within SD trials. For baseline estimates, we computed $P_{\text{control}}$ from a single 300-ms window spanning −300 to 0 ms (centered at −150 ms). Time-resolved SD activity was then estimated in 300-ms windows whose centers advanced in 10-ms steps from −300 to 1800 ms, and the SD > Baseline contrast used $P_{\text{SD}}$ (t) from each SD window relative to this fixed prestimulus baseline.

Time-locked control (for SD > SYM). The SYM control used time-locked SYM epochs with the same 300-ms window length and 10-ms step, aligned to stimulus onset. The SD > SYM contrast used $P_{\text{control}}$ from SYM windows and $P_{\text{SD}}$ from SD windows, with the same common spatial filter applied to both conditions.

For LI analyses, peak-based summaries were constrained to windows whose centers lay between 300 and 1200 ms post-stimulus, to avoid residual cognitive and motor activity from the preceding trial and early sensory responses while encompassing the main beta-suppression epoch. Participant/ROI-specific 300-ms windows were then selected within this range using a bound-optimized procedure based on peak asymmetry in regional response magnitude (see LI Dynamics).

#### 2.3.5. Regions of interest (ROIs)

**Fig. 2.**
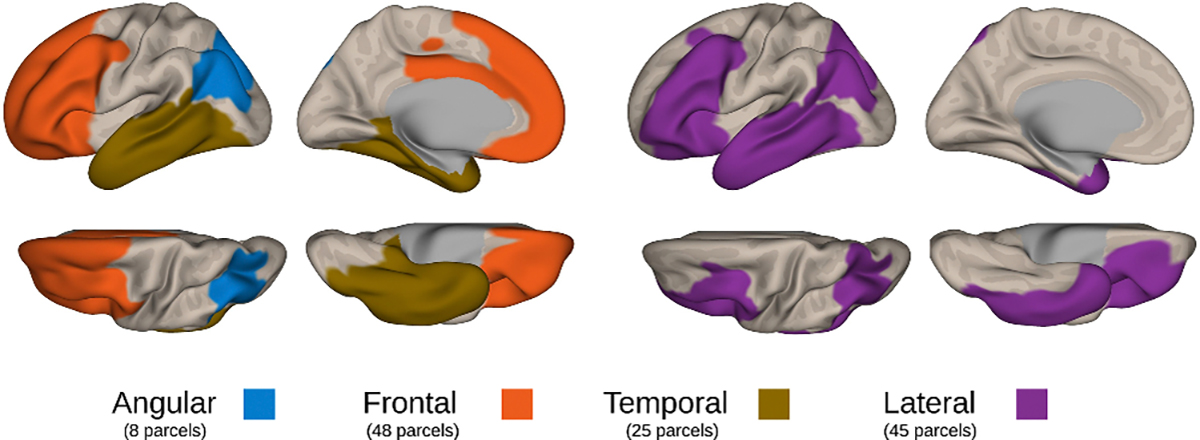
Cortical masks based on the HCP-MMP1.0 multimodal parcellation. Surface renderings show the four ROIs used for source-level laterality analyses generated by combining HCP-MMP 1.0 parcels. Left: three canonical language-network masks—Angular gyrus (blue), Frontal language cortex (orange), and Temporal language cortex (olive). Right: an aggregated Lateral mask (purple) pooling language-relevant parcels across lateral frontal, lateral temporal, and angular gyrus regions. For clarity, only the left hemisphere is displayed; right-hemisphere homologues were defined from the corresponding HCP-MMP parcels and used symmetrically in all analyses.

Cortical regions of interest (ROIs) were defined using the HCP-MMP1.0 atlas (Glasser et al., 2016). Parcels were grouped into four language-related ROIs based on activation to the SD–TD contrast in previous fMRI studies. A Frontal ROI (48 parcels) included inferior frontal, middle frontal, superior frontal, and lateral/orbital frontal regions. A Temporal ROI (25 parcels) included superior temporal, middle/lateral temporal, ventral temporal, and medial temporal parcels. An Angular gyrus ROI (8 parcels) covered the posterior inferior parietal cortex surrounding the angular gyrus, including the adjacent temporo-parietal-occipital junction and dorsal parietal parcels. A Lateral ROI (45 parcels) comprised the union of lateral frontal, lateral temporal, and inferior parietal (angular/temporoparietal junction) parcels. The Lateral ROI was defined a priori to mirror the distributed language mask used in prior fMRI validation studies (e.g., Binder et al., 2008a; Gross et al., 2025, 2022; Janecek et al., 2013a), enabling direct cross-modal LI comparison. The spatial layout is shown in Fig. 2. These ROIs were defined a priori to preserve direct comparability with the matched fMRI laterality masks and were not modified post hoc on the basis of the current MEG activation maps. Accordingly, task-responsive regions outside these masks, such as ventral sensorimotor or non-prespecified inferior parietal activity, were not included in the primary LI analysis. For brevity, Table S2 lists hemisphere-agnostic HCP parcel codes; analyses used left and right homologues.

#### 2.3.6. Laterality index (LI)

To quantify hemispheric dominance, we computed the laterality index $LI=\frac{L-R}{L+R}$, where L and R are the relevant activations in the left- and right-hemisphere ROIs, respectively. We implemented three LI variants based on how the relevant activation was defined at each ROI: suprathreshold magnitude summation, suprathreshold vertex counting, and a weighted bootstrap approach adopted from the fMRI literature, to capture complementary aspects of hemispheric activity.

Suprathreshold magnitude summation LI: We identified cortical sources exceeding a single threshold per participant × ROI, defined as 50% of the global maximum regional source value across all intervals (see **§**2.5.1). The LI was then computed from the sum of suprathreshold magnitudes in each hemisphere, emphasizing strongly responsive vertices.

Vertex counting LI: Using the same threshold criterion, we counted the number of suprathreshold vertices per hemisphere within each ROI, emphasizing the spatial extent of activation.

Weighted bootstrap LI: Following Wilke and Schmithorst (2006), we used a multi-threshold, resampling-based LI. For each participant × ROI, we drew 300 bootstrap resamples (75% of vertices with replacement) to form a distribution of LI values across a grid of 20 thresholds spanning the observed range [*T*_min_, *T*_max_]. We selected 300 iterations for computational efficiency after a convergence check on a subset of subjects/ROIs showing that increasing to 1000 resamples altered the median LI by < 0.01 and the CI width by < 0.02, while tripling runtime. Source maps were summarized in 300-ms windows advanced every 10 ms from −300 to 1800 ms (see Task contrasts). For each bootstrap i, a threshold-weighted LI was computed as,

$${\text{Combined\_LI}}_{i}=\left(\frac{\sum_{t=1}^{N_{T}}\;LI_{t}^{\left(i\right)}\cdot t}{\sum_{t=1}^{N_{T}}\;t}\right)\times 100$$

where ${LI}_{t}(i)$ is the LI at threshold t for resample i and $N_{T}=20$. The participant’s LI estimate is the median of ${\text{Combined\_LI}}_{i}$; 95% CIs are the 2.5th/97.5th percentiles. Categorical labels were left if LI > +10, right if LI < −10, and otherwise symmetric.

By employing these three methods—source magnitude, vertex counting, and weighted bootstrap—our objective was to capture both the intensity and spatial extent of task-related hemispheric activations.

### 2.4. fMRI Data analysis

Preprocessing used standard steps in FSL/AFNI: removal of the first four volumes, slice-timing correction, rigid-body realignment with six degrees of freedom, distortion correction with fieldmaps, coregistration of the mean EPI image to the T1-weighted anatomical image, normalization to MNI-152 space at 2-mm isotropic resolution, spatial smoothing with a 6-mm FWHM Gaussian kernel, and temporal high-pass filtering with a 128-s cutoff.

All runs were modeled jointly in a single general linear model using 3dREMLfit in AFNI. The SD and TD conditions were entered as separate regressors and convolved with a one-parameter gamma-variate hemodynamic response function. Nuisance regressors included the six motion parameters, their temporal derivatives, and mean cerebrospinal fluid and white matter time series. Framewise displacement was computed for each volume, and volumes with FD > 0.9 mm were censored. The primary contrast of interest was SD > TD. Laterality indices were derived from the resulting SD > TD t-maps using the same ROIs as the MEG analysis: Frontal, Temporal, Angular, and Lateral. fMRI LIs were computed using an analogous weighted-bootstrap procedure based on Wilke and Schmithorst (2006), with thresholds of t = 1.5:0.1:4.0, 1000 resamples, the median as the LI estimate, and the 2.5th and 97.5th percentiles as the 95% confidence interval. Dominance labels were defined as left if LI > +10, right if LI < −10, and symmetric otherwise, harmonized with the MEG analysis.

### 2.5. MEG–fMRI Concordance

We assessed concordance between MEG and fMRI within the Frontal, Temporal, Angular, and Lateral ROIs. Agreement was defined as matching categorical labels (left/right/symmetric) between modalities. For summary metrics, we also computed (i) the continuous difference between MEG and fMRI LI |LI_MEG_–LI_fMRI_| and (ii) a ternary code for dominance (+1 = left, 0 = symmetric, −1 = right) with the corresponding absolute ternary difference |trn_MEG_–trn_fMRI_|. Codes were used only for summary statistics; categorical labels (left/right/symmetric) were determined from bootstrap LIs. Pearson correlations between continuous LIs were computed per ROI, and window-wise concordance (%) was computed across 300-ms windows stepped every 10 ms within the 300–1200 ms analysis range.

#### 2.5.1. LI Dynamics: fixed windows vs. participant/ROI-specific windows

To test a method for refining MEG–fMRI concordance, we used two windowing strategies for LI computation.

Fixed window (cohort). We identified a single 300-ms temporal window within the 300–1200 ms period after stimulus onset that maximized MEG–fMRI concordance across all participants (for each ROI and contrast family) and used that same window for all participants.

##### Participant/ROI-specific window.

To identify candidate time points for each participant × ROI, we defined the regional response index (rRI) as the hemispheric sum of beta-suppression magnitudes within the ROI. For each participant × ROI, we computed

$$\Delta rRI(t)=\left|rRI_{L}(t)-rRI_{R}(t)\right|$$

at every time point between 300 and 1200 ms. We then took the 95th percentile of this within-participant, within-ROI distribution of ΔrRI(t) values (P95) and set a threshold θ=0.05×P95. Time points with ΔrRI(t)≥θ were considered candidates for centering the 300-ms analysis window. Among these candidate time points, we selected the one with the largest ΔrRI(t) and centered the 300-ms window there. If multiple time points shared the maximum value, we chose the earliest; if no time point exceeded the threshold, we used the global maximum within 300–1200 ms. An example is shown in Fig. 6, and trajectories for all participants are shown in Fig. S1.

To keep LI values comparable over time, both the source-magnitude and vertex-count methods used one fixed threshold per participant × ROI: 50% of that ROI’s maximum source value across all intervals. The same threshold was used for fixed and participant/ROI-specific windows.

### 2.6. Cross-ROI stability of lateralization indices

To quantify within-modality stability of hemispheric dominance across ROIs, we examined both categorical and continuous cross-ROI relationships within MEG and within fMRI laterality measures.

For the categorical analysis, we used the ternary LI coding described above (left-dominant: LI ≥ +10; bilateral: −10 < LI < +10; right-dominant: LI ≤ −10). Within each modality separately (MEG or fMRI), we asked for each patient whether at least one ROI was classified as left-dominant and at least one other ROI as right-dominant. Such cases were labeled as showing a “cross-ROI reversal” of dominance. We report the proportion of patients with at least one left↔right reversal for MEG and fMRI and compared these paired proportions using McNemar’s chi-square test on the 2 × 2 table of reversal vs non-reversal across modalities.

For the continuous analysis, we used the subject-wise continuous LIs (MEG: optimal beta band LI per ROI; fMRI: ROI-wise LI from the semantic-decision task) and constructed, separately for each modality, a 4 × 4 ROI–ROI correlation matrix (Angular, Frontal, Temporal, Lateral). Pearson correlation coefficients were computed across subjects for each ROI pair, using pairwise deletion for missing values. To summarize cross-ROI stability, we calculated the mean off-diagonal correlation (averaging the six unique ROI pairs) for each modality. The difference in cross-ROI stability between MEG and fMRI was assessed by Fisher-z transforming the six ROI–ROI correlations per modality and performing a paired *t*-test on the z-values (MEG vs fMRI) across ROI pairs.

### 2.7. Predictors of MEG–fMRI discordance

To identify factors associated with disagreement between MEG and fMRI LIs, we examined 17 candidate predictors/covariates spanning five domains: behavioral performance, epilepsy severity, cognitive function, MEG signal quality, and response SNRs (Table S3). Discordance was quantified both as the absolute LI difference (|MEG – fMRI|) and the absolute ternary difference (|MEG_trn – fMRI_trn|). Binary concordance status (concordant vs. discordant) was determined using bootstrap LI criteria: left if LI > +10, right if LI < −10, and symmetric otherwise.

Because partial cases (one modality clearly lateralized, the other symmetric) typically arise when one modality’s point LI lies near the ±10% neutral zone (often with wide confidence intervals), they are susceptible to small, non-systematic shifts and can dilute associations. We therefore focused predictor analyses on gross discordance (left↔right reversals with both modalities clearly lateralized by the LI criterion, |LI| ≥ 10), which is clinically consequential and less confounded by near-neutral LIs. Overall concordance statistics still include all cases; covariate screens target gross discordance to maximize specificity.

The behavioral domain included mean reaction time and accuracy for the semantic-decision and symbol matching tasks. Clinical epilepsy burden was indexed by seizure-focus laterality (TLE side: left, right, or bilateral/uncertain), the number of concurrent antiepileptic drugs (AEDs), and patient-reported seizure measures: complex-partial seizure frequency (CP_freq), simple-partial seizure frequency (SP_freq), secondarily generalized seizure frequency (SG_freq), and lifetime generalized tonic–clonic seizure count (LTGTC). Handedness was recorded with the Edinburgh Handedness Inventory (EHI). Overall cognitive function was measured by full-scale IQ (FSIQ; WASI-II). Signal quality was assessed by (i) regional response index rRI, the larger hemispheric sum of beta-suppression magnitudes within the ROI showing peak suppression, and (ii) artifact-suppression gains in beta and broadband ranges across processing steps (raw → tSSS; tSSS → MEGnet).

For univariate analyses, we correlated predictors with the two continuous discordance measures using Pearson’s r for |MEG – fMRI| and Spearman’s ρ for |MEG_trn – fMRI_trn| (which has a discrete/ordinal range). Associations with binary concordance were evaluated using point-biserial correlations. We controlled p-values using Benjamini–Hochberg FDR (α = 0.05). In a complementary analysis, we discretized 14 variables (excluding rRI and the two LI-difference covariates) and tested associations with binary concordance using Fisher’s exact tests (2 × 2 or 2 × 3), summarized in Tables S4–S5.

Categorization cutoffs were based on prior literature and inspection of the empirical distributions: Accuracy (<50%, 50–80%, >80%); Reaction time (<1.0 s, 1.0–1.5 s, >1.5 s); FSIQ (<80, 80–120, >120); AED load (0, 1, 2, ≥3). Patient-reported seizure measures were binned into 2–3 levels (exact cutpoints in Table S4). SNR gains (artifact-suppression gain in beta and broadband across processing steps: raw → tSSS; tSSS → MEGnet) were similarly binned. The regional response index rRI (largest hemispheric sum of beta suppression within the ROI showing peak suppression) was retained as a continuous predictor.

For multivariable analysis, we fit a Firth logistic regression with concordance status as the outcome. The top three FDR-ranked continuous predictors entered as z-scored fixed effects; categorical predictors were dummy-coded. Given the small number of grossly discordant cases, this model served primarily for robustness checking rather than formal hypothesis testing.

## 3. Results

### 3.1. Task performance

Participants performed both tasks quickly and accurately. In the semantic-decision (SD) blocks, mean RT was 1.17 ± 0.15 s with 79.3 ± 11.9% accuracy; the symbol-matching control (SYM) was slightly slower (1.29 ± 0.17 s) but more accurate (87.2 ± 11.9%). Within each task, faster responses predicted higher accuracy (SD: r=−0.41, p = 4.0 × 10^−4^; SYM: r=−0.54, p = 1.8 × 10^−6^).

### 3.2. Group beta band suppression maps

**Fig. 3.**
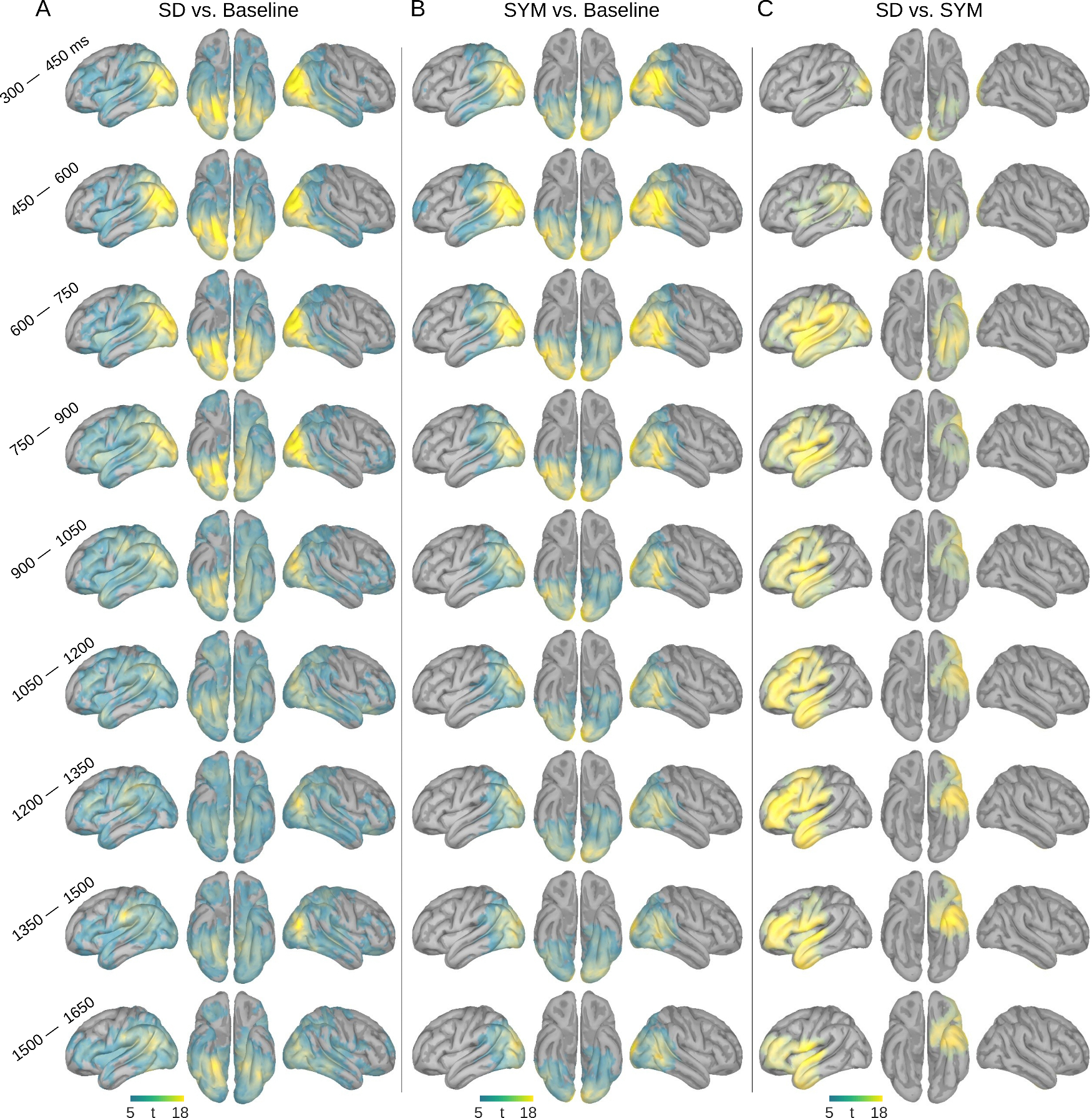
Time-resolved beta-band source-power modulation for the semantic-decision and symbol-matching tasks. (A-B) Group-level cortical maps show beta-band source-power changes in the 17–25 Hz range across successive 300-ms analysis windows centered at the latencies indicated on the left. The left and middle columns show task-related beta modulation for the semantic-decision (SD) and symbol-matching (SYM) conditions, respectively, relative to the prestimulus baseline. (C) The right column shows the direct SD > SYM contrast, highlighting language-related beta modulation beyond activity shared by the visual/control task. Warmer colors indicate stronger task-related effects, with maps thresholded and displayed as t-statistics. Across time, both tasks show evolving posterior and temporal beta modulation, whereas the direct contrast reveals more left-lateralized temporal–frontal involvement during the semantic-decision condition. (For interpretation of the references to color in this figure legend, the reader is referred to the web version of this article.)

Fig. 3 shows stimulus-locked 17–25 Hz beta-band source-power suppression assessed with a one-sided cluster-based permutation test using a cluster-forming threshold of t < −5, 1000 randomizations, and FWE-corrected cluster p < 0.01. Maps are shown in representative post-stimulus time windows from 300 ms to 1650 ms after stimulus onset.

In the SD > Baseline contrast (Fig. 3A), bilateral beta suppression involves lateral and ventral occipital cortex and posterior ventral temporal cortex from the earliest time window and persists throughout much of the trial. These occipitotemporal responses show rightward lateralization after approximately 750 ms. A weaker, left-lateralized response is also observed from 300 ms onward, involving lateral parietal cortex, insula, and lateral frontal cortex. From 600–1200 ms, this left-lateralized response becomes progressively stronger and more broadly distributed, including a prominent response in the left anterior temporal lobe peaking around 1050–1200 ms. A weaker response in the right lateral frontal cortex, centered in the middle frontal gyrus, and right anterior insula occurs from 750 to 1500 ms. From 1200–1500 ms, responses progressively diminish, followed by a late increase in the 1500–1650 ms window, particularly in left posterior parietal cortex, including angular gyrus, and bilateral ventral occipitotemporal cortex.

In the SYM > Baseline contrast (Fig. 3B), beta suppression is also evident across the post-stimulus interval. Early responses are observed bilaterally in posterior occipital and ventral occipitotemporal cortex. Compared with SD > Baseline, the SYM > Baseline contrast shows less prominent left-lateralized involvement of frontal, temporal, and inferior parietal regions. Across later windows, SYM-related suppression remains most evident in posterior occipital and ventral temporal regions, with more limited extension into lateral frontal and anterior temporal regions.

In the direct SD > SYM contrast (Fig. 3C), effects are more spatially selective and more strongly left-lateralized than in either baseline contrast. An early effect from 300 ms mainly involves left lateral occipital cortex, then extends into left angular and supramarginal gyri by 450 ms and more broadly into insula, temporal cortex, pre- and post-central gyri, and inferior and middle frontal gyri by 600 ms. From approximately 900 ms onward, the frontal response also includes superior frontal cortex and frontal pole. The temporal lobe response begins early in left parahippocampal and fusiform regions, then spreads to broader left temporal cortex by 600 ms. After 750 ms, occipital and posterior parietal responses diminish, whereas left frontal, pre/post-central, insular, and temporal responses persist, with later temporal effects becoming more focused in the anterior temporal lobe and temporal pole.

### 3.3. Sliding-window laterality and concordance

**Fig. 4.**
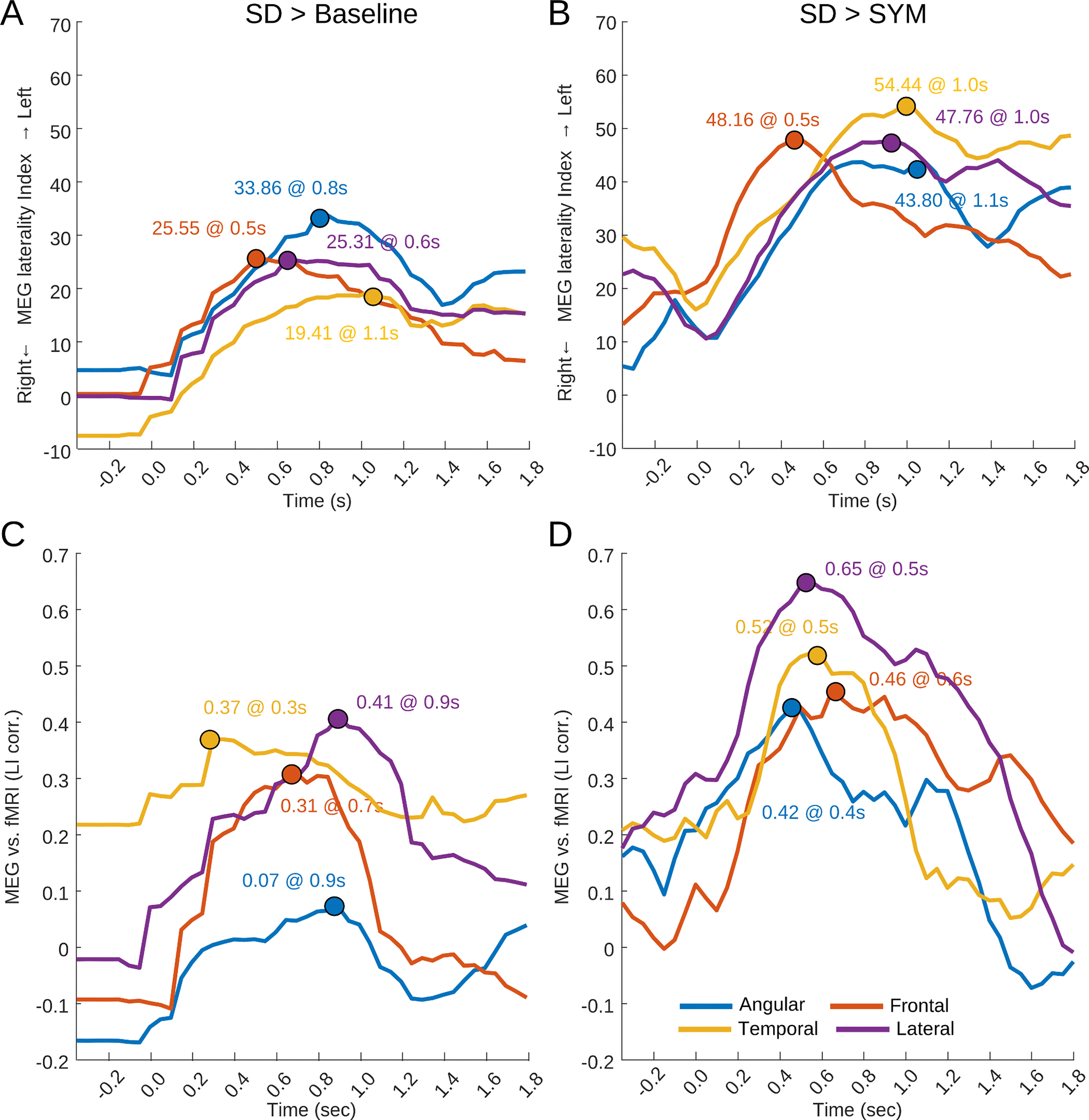
Temporal evolution of language laterality and correlation with fMRI. (A) Grand-average MEG laterality indices (LI; positive = left) for SD > Baseline, estimated with a 300-ms sliding window advanced by 10 ms from −0.30 to 1.80 s relative to stimulus onset. (B) Same analysis for SD > SYM. Curves are color-coded by ROI: Angular (blue), Frontal (orange), Temporal (yellow), and Lateral composite (purple); filled circles mark each ROI’s peak LI and its latency. (C) Running Pearson correlation between the SD > Baseline MEG LI trajectories in (A) and participant-specific fMRI LIs in matched ROIs. (D) Corresponding correlation for SD > SYM LIs in (B). In the grand-average LI trajectories across 74 TLE participants, Frontal LI peaked earliest at approximately 0.5 s, whereas the Lateral composite, Temporal, and Angular ROIs reached their strongest leftward lateralization later, particularly in the SD > SYM contrast. Confidence bands have been omitted for clarity. (For interpretation of the references to color in this figure legend, the reader is referred to the web version of this article.)

Fig. 4A and B show time-resolved laterality indices (LIs; 300-ms windows advanced every 10 ms), averaged over the entire cohort, and their running Pearson correlation with fMRI LIs for the Frontal (orange), Temporal (yellow), Angular (blue), and Lateral (purple) ROIs. LIs for this figure were computed using the multi-threshold bootstrap method.

Under SD > Baseline (Fig. 4A), LI peaks first in the frontal cortex (+25.6 at 500 ms), followed by Lateral (+25.3 at 600 ms), Temporal (+19.4 at 800 ms), and Angular (+33.9 at 1100 ms). Peak MEG–fMRI correlation (Fig. 4C) reached *r* = 0.41 in the Lateral ROI at 900 ms, while Angular correlations remained < 0.10.

With SD > SYM (Fig. 4B), LIs were more strongly lateralized but peaked in a similar or slightly later time frame: Frontal reached +48.2 at 500 ms; Lateral peaked at +54.4 at 950 ms; Temporal and Angular rose nearly in parallel (+47.8 at 1000 ms and +43.8 at 1100 ms, respectively). Correlations between MEG and fMRI LIs were also higher, with a maximum of *r* = 0.65 in the Lateral ROI at 500 ms (vs. 0.41 under SD > Baseline; Δr = 0.24); peak correlations in the other ROIs were *r* = 0.42 (Angular, 400 ms), *r* = 0.52 (Temporal, 500 ms), and *r* = 0.46 (Frontal, 600 ms).

**Fig. 5.**
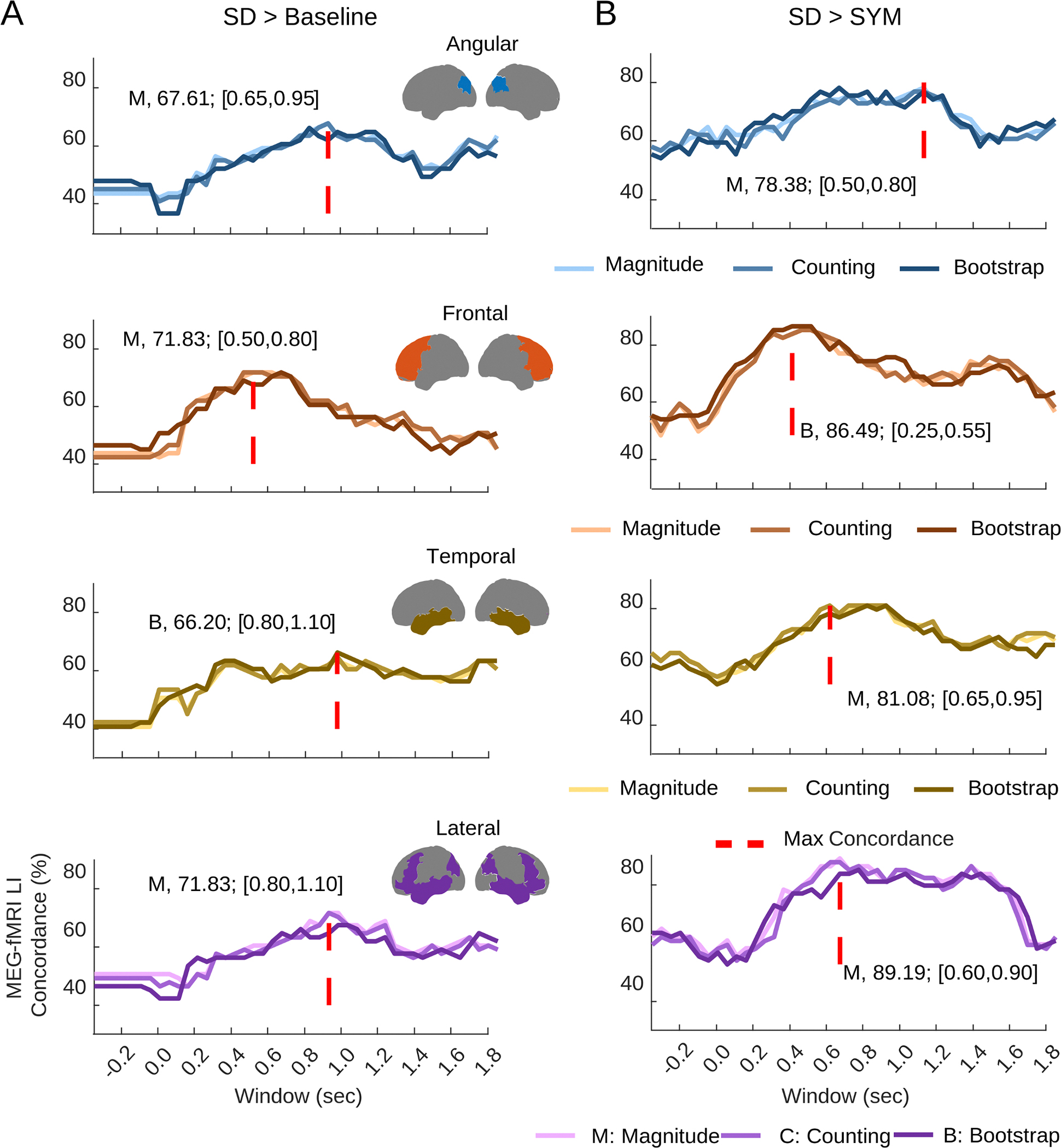
Window-wise concordance of MEG–fMRI ternary language-laterality classifications across LI metrics, ROIs, and contrasts. For each ROI, Angular, Frontal, Temporal, and Lateral composite, curves show the percentage of participants whose MEG LI classification matched the fMRI classification across 300-ms windows advanced in 10-ms steps. Three LI schemes are overlaid: magnitude, counting, and bootstrap. (A) SD > Baseline. (B) SD > SYM. Red dashed arrows indicate the peak-concordance window for the best-performing LI metric in each panel; labels report the metric, peak concordance, and the contiguous interval over which concordance remained within 95% of that peak. Relative to SD > Baseline, the symbol contrast generally increased concordance, most notably in the Lateral composite ROI, where magnitude LI reached approximately 89% around 0.6–0.9 s. The symbol contrast also shifted the optimal window earlier in the Frontal ROI and later in the Temporal ROI. Confidence bands are omitted for clarity.

Fig. 5 summarizes categorical agreement with fMRI across windows for each LI algorithm, using a ternary classification (left, right, symmetric). With a prestimulus baseline as control, agreement plateaued at around 72% in Frontal and Lateral ROIs and slightly lower in Angular and Temporal ROIs. The symbol control elevated concordance across ROIs, yielding 86% in Frontal (bootstrap LI), 78% in Angular, 81% in Temporal, and a study-wide maximum of 89% in the Lateral ROI. Differences in concordance between the three LI methods were relatively minor, and no method produced uniformly higher values.

Together, these trajectories indicate that frontal LI peaked earlier than temporal–parietal LI, whereas lateral, temporal, and angular ROIs reached their strongest leftward lateralization later in the post-stimulus time course. Incorporating the symbol-matching task as a control condition increased LI magnitude, MEG–fMRI correlations, and categorical MEG–fMRI concordance (Figs. 4–5). The highest concordance was obtained using the SYM task control and Lateral ROI, and a temporal window of 600–900 ms.

### 3.4. Participant/ROI-specific LI analysis

**Fig. 6.**
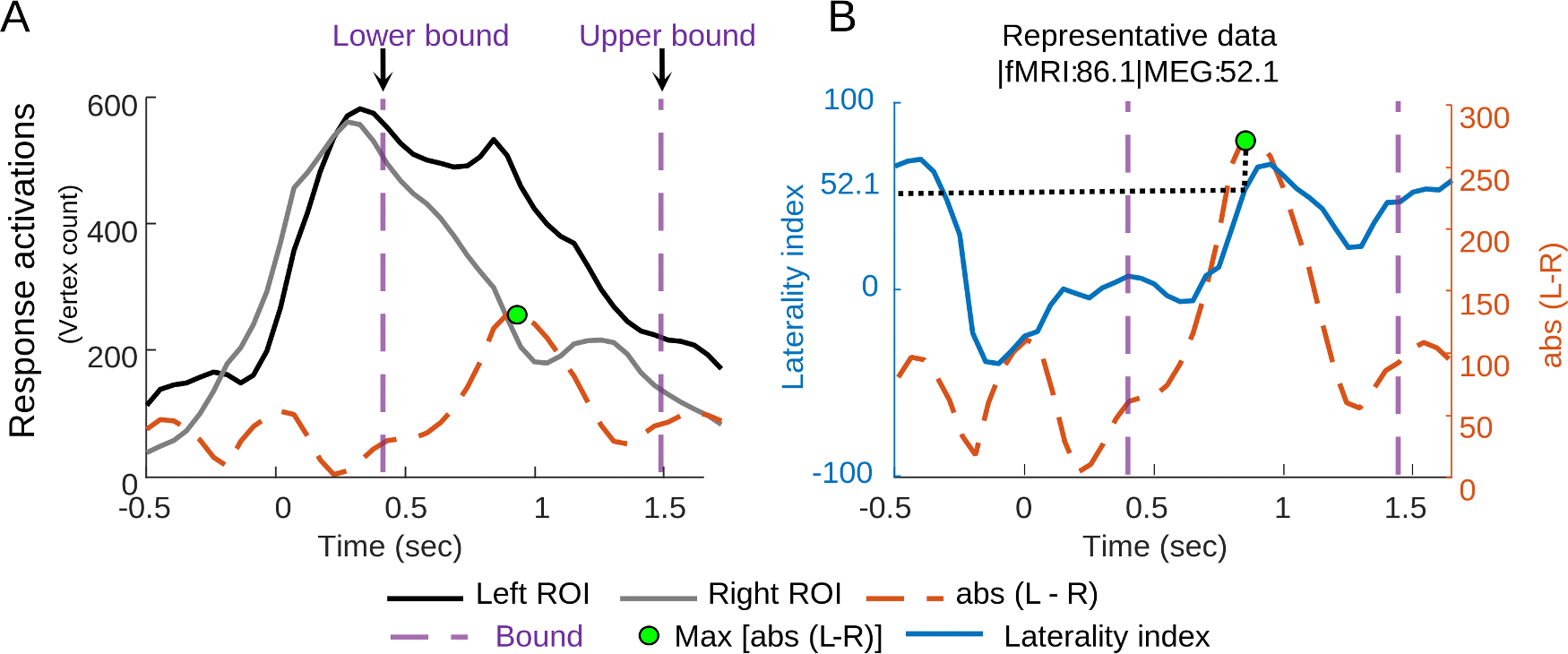
Method for determining participant-specific windows that maximize MEG–fMRI concordance. (A) Regional Response Index (rRI)-guided, bound-optimized peak selection (representative participant/ROI). Hemisphere traces rRI_L(t) and rRI_R(t) and the decision signal ΔrRI(t) = |rRI_L(t) – rRI_R(t)| (orange line) are computed in 300-ms moving windows advanced every 10 ms for SD > SYM within 300–1200 ms. The horizontal line indicates the subject-specific threshold θ = 0.05 × P95(ΔrRI); high-rRI bounds (purple, dashed) are the first/last threshold exceedances. (B) The bootstrap LI(t) (blue) is shown with the selected ΔrRI peak center time (green circle) representing the 300-ms analysis window used in panel B and Table 2. See Fig. S1 for all participants’ trajectories.

**Fig. 7.**
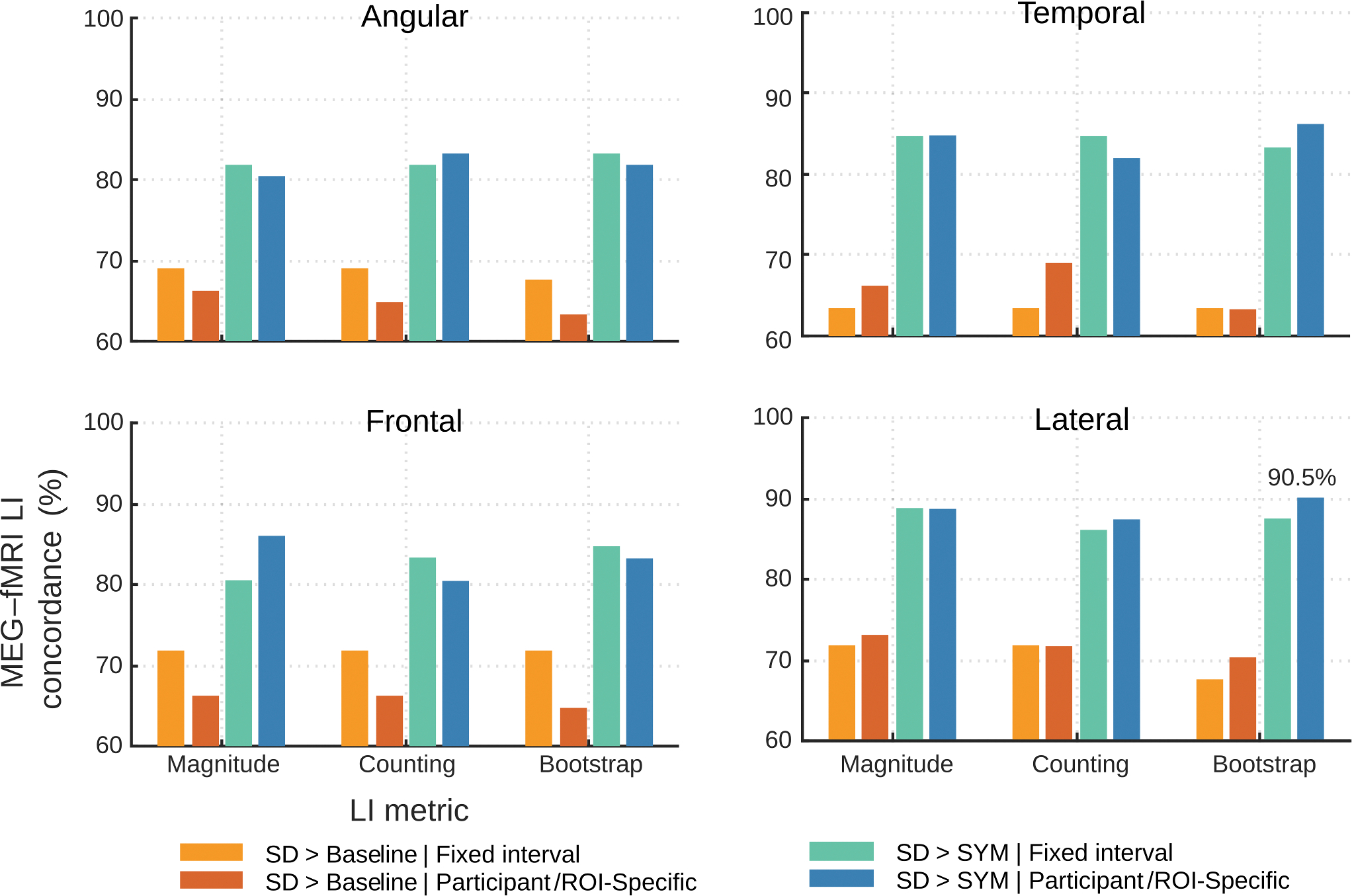
Comparison of MEG–fMRI language-laterality concordance by LI metric, windowing strategy, and contrast. For each LI metric—source magnitude, vertex counting, and weighted bootstrap—bars show the percentage of 74 TLE participants whose MEG LI category (left/right/symmetric) matched the fMRI category. Within each metric, four variants are shown: (i) SD > Baseline in a fixed cohort 300-ms window chosen within 0.30–1.20 s, (ii) SD > Baseline at each participant’s 300-ms peak window, (iii) SD > SYM in the fixed cohort window, and (iv) SD > SYM at the participant-specific peak window. Replacing the prestimulus baseline with the symbol control increased concordance, whereas tailoring the window to individual peak activity produced small and mixed effects across ROIs and LI metrics. The highest agreement, 67/74 participants (90.5%), was achieved with the weighted-bootstrap LI for SD > SYM in participant-specific peak windows in the Lateral ROI. Panels show Angular and Temporal ROIs in the top row and Frontal and Lateral ROIs in the bottom row.

Fig. 6 illustrates the method used for selection of patient-specific time windows for LI measurement. Peak beta-suppression latency varied by ROI and participant (e.g., 350–1200 ms in the Lateral ROI; Fig. S1). Using the SD > SYM contrast and a 300-ms window centered on each participant’s peak left–right difference produced modest gains in percent concordance in the Temporal (+2.8%) and Lateral (+2.7%) ROIs, with no improvement in the Frontal ROI and a slight decline in the Angular ROI (−1.4%) (Fig. 7). Combining both optimizations (SYM control + participant-specific timing) yielded 86% concordance in Frontal, 81% in Temporal, 78% in Angular, and 90% in the Lateral ROI using the bootstrap LI (Fig. 7; Table 2).

### 3.5. Cross-ROI stability of MEG and fMRI LIs

To assess within-modality stability of hemispheric dominance, we examined how often patients changed dominance category across ROIs. For MEG, 20/74 patients (27.0%) showed at least one left-dominant and one right-dominant ROI, compared with 7/74 (9.5%) patients for fMRI. This difference in cross-ROI left↔right “reversals” was significant (McNemar χ^2^(1)=6.26, p = 0.012). Using continuous LIs, we next computed ROI–ROI correlations within each modality. MEG LIs showed moderate cross-ROI correlations (mean off-diagonal $r^{-}=0.45$), whereas fMRI LIs were highly correlated across ROIs (mean off-diagonal $r^{-}=0.81$). A paired test on Fisher-z–transformed correlations confirmed higher cross-ROI stability for fMRI than MEG (p = 0.0017). These findings indicate that MEG beta band lateralization exhibits greater regional variability across the language network than fMRI, even though MEG–fMRI concordance remains high in the Lateral ROI used as our primary clinical readout.

### 3.6. Residual discordance

MEG and fMRI identified the same language-dominant hemisphere in 67/74 participants (90.5%; Cohen’s κ = 0.81). Among the seven discordant cases, five were gross discordances (left↔right reversals with opposite-signed LIs) and two were partial discordances, where one modality was clearly lateralized and the other was near symmetric (e.g., IDs 20 and 57 in Fig. S2). In the Lateral ROI, gross reversals included IDs 10 and 28 (MEG ≈ −55/−50; fMRI ≈ +60), 4 (MEG ≈ +45; fMRI ≈ −60), and 5 (MEG ≈ −60; fMRI ≈ +25). The scatterplot displays in Fig. S2 show both gross (orange points) and partial (yellow points) discordances.

To probe potential drivers of gross discordance, we evaluated behavioral, clinical, cognitive, and data-quality covariates using continuous correlations (Fig. S3) and categorical Fisher tests (Tables S4–S5), including clinical factors such as side of seizure onset (left vs. right), epilepsy duration, seizure frequency, and AED load. No categorical comparison (2 × 2 or 2 × 3) survived FDR control (q < 0.05); the closest trend was SD task accuracy in the Angular ROI (q = 0.08). In the correlation heatmaps, we did not observe a robust, FDR-significant predictor of the absolute MEG–fMRI LI difference across ROIs. The only notable covariate–LI association was a mild negative correlation between AED load and MEG LI in the Angular ROI; otherwise, panels showed the expected internal relationships among the LI measures (MEG LI positively correlated with fMRI LI; |MEG – fMRI| decreased as either of the LIs strengthened; the signed MEG – fMRI difference correlated in opposite directions with MEG vs. fMRI LI). Side of seizure onset did not show any detectable association with discordance rates or |MEG–fMRI| differences. A bias-reduced (Firth) logistic regression including the three highest-ranked continuous predictors after FDR correction (all z-scored), fMRI_LI, optMEG_LI, and absolute MEG–fMRI LI difference, did not reveal significant associations with discordance (all p > 0.05), although statistical power was limited by the small number of discordant cases (*n* = 7).

Taken together, the residual mismatches in this cohort are dominated by cross-hemisphere reversals; we did not identify a consistent behavioral, clinical, cognitive, or signal-quality predictor of discordance across ROIs under the tested models.

## 4. Discussion

### 4.1. Principal findings and contribution

In a large single-center cohort of adults with TLE, we found that beta band (17–25 Hz) MEG power suppression during a SD task can provide language-dominance estimates that show high agreement with fMRI when three methodological choices are combined. These choices are (i) use of a contrast against a nonlinguistic control task rather than a prestimulus baseline, (ii) participant/ROI-specific peak windows instead of a single canonical bin, and (iii) a threshold-robust weighted-bootstrap LI with confidence intervals. Together, these choices yielded 90.5% overall agreement between MEG and fMRI ternary dominance labels (67 of 74 participants; κ = 0.81) in a lateral hemisphere ROI that spans frontal, temporal, and angular cortices (Figs. 4–7). These findings should be interpreted as evidence of strong MEG–fMRI concordance of hemispheric language dominance estimates rather than direct validation of MEG against a ‘gold standard’.

The effects of these choices were variable. The nonlinguistic task control greatly increased the magnitude of power changes and LIs in all ROIs, advanced the timing of peak lateralization, and substantially increased the level of concordance between MEG and fMRI. rRI-guided peak centering captured inter-individual and regional variability in response latency but had little consistent effect on MEG–fMRI concordance (Fig. 7). The weighted-bootstrap LI produced broadly similar MEG–fMRI concordance to magnitude- and vertex-count-based metrics, with only small, mixed differences across ROIs and contrasts. Beyond agreement with fMRI, the data reveal a physiologically coherent pattern of left-hemisphere lateralization of the SD > SYM differential response, with earlier frontal lateralization followed by later temporal and parietal lateralization, consistent with language-related processing rather than generic motor or arousal effects (Fig. 3).

### 4.2. Spatiotemporal cascade and task specificity

Stimulus-locked beta (17–25 Hz) suppression maps (Fig. 3) index response strength and reveal a reproducible cascade of cortical engagement: early bilateral decreases in occipito-temporal regions associated with visual word-form processing (approximately 300–450 ms) (Dehaene and Cohen, 2011), followed by widespread engagement of lateral temporal, parietal, and frontal cortex. By 600–900 ms, this network becomes clearly left-dominant, encompassing the left lateral frontal (including inferior and middle frontal gyri), anterior and posterior temporal, and inferior parietal cortices, which have been implicated in lexical selection, semantic integration, and decision processes (Binder et al., 2009; Indefrey and Levelt, 2004; Price, 2012).

With the symbol-matching control (SD > SYM), this left-dominant pattern emerges earlier and more strongly than with a prestimulus baseline, with peaks around 750–900 ms and sustained effects to approximately 1.5–1.65 s. The added SYM vs. baseline maps in Fig. 3B further show that the control task itself elicits robust, temporally evolving beta suppression, particularly in posterior occipital and ventral occipitotemporal regions. Thus, the earliest SD > SYM window (150–450 ms, centered at 300 ms) should not be interpreted as subtraction against a stationary visual-only SYM state. Rather, it reflects a matched early comparison between two rapidly evolving tasks, both dominated at this stage by visual/perceptual processing and both preceding overt behavioral responses. Illustrative single-subject sensor-level TFRs are shown in Supplementary Fig. S4.

Importantly, the SYM condition is not assumed to be a static or purely sensory baseline. The slightly longer reaction time in SYM than in SD suggests that the two tasks have distinct temporal dynamics as the trial unfolds. The SD > SYM maps were nevertheless computed from matched, time-locked windows using the same common spatial filter applied to both conditions, and are therefore best interpreted as differential-response maps in which shared visual/perceptual and time-locked response-related components are reduced while net task-related differences in beta suppression are emphasized. Thus, early windows likely reflect common visual processing plus the earliest divergence between tasks, whereas later windows reflect the evolving balance between lexical-semantic/decision-related recruitment in SD and nonlinguistic matching/decision processes in SYM, rather than a subtraction of “language” from a stationary visual baseline.

The sliding-window LIs (Fig. 4) emphasize hemispheric asymmetries rather than absolute response amplitude and therefore show a somewhat different sequence from the activation maps. In the source maps (Fig. 3), temporo-parietal regions are among the first to show robust beta suppression, with largely bilateral engagement early in the epoch. In the LI trajectories, however, the left lateral frontal cortex shows a clear leftward shift slightly earlier than the temporal and angular ROIs. In other words, the frontal cortex is not the first region to become active, but it is the first to exhibit a pronounced hemispheric bias in beta suppression. Large-scale neuroimaging and connectivity work, including a meta-analysis of 403 language experiments (Turker et al., 2023), highlights the left inferior-middle frontal cortex as a core hub within the fronto-temporal language system. Task-based studies emphasize that contributions of the left lateral frontal cortex to language depend on task demands and individual ability, consistent with a control component layered on top of linguistic processing (Badre et al., 2005; Jackson, 2021; Katzev et al., 2013; Novick et al., 2005; Thompson-Schill et al., 1997). Gradient-based functional connectivity analyses further suggest a graded organization within the left lateral frontal cortex, with more dorsal portions more strongly linked to domain-general control networks and more ventral portions more tightly coupled to lexical–semantic and phonological networks (Diveica et al., 2023). Within this framework, the slightly earlier and stronger leftward lateralization in the frontal ROI can be interpreted as an emerging left frontal control bias within an already engaged, partly bilateral temporo-parietal representational system, rather than as strictly serial “frontal then temporo-parietal” engagement.

Together with motor/visual matching by the symbol control, these patterns support a language-specific interpretation of the beta power suppression effects, reflecting controlled lexical-semantic processing and decision-making, rather than nonspecific arousal or motor output (Kilavik et al., 2013; Singh et al., 2002).

### 4.3. Why the nonlinguistic control task improves lateralization

Replacing a prestimulus baseline with a matched nonlinguistic control reduces variance related to low-level visual, oculomotor, vigilance, and button-press components that are shared by words and false fonts, thereby increasing contrast-to-language signal and reducing bilateral confounds (Binder et al., 2008b; Seghier, 2008). In our cohort, subtracting the symbol condition increased response magnitude and LI magnitude, and improved both continuous and categorical correspondence with fMRI (Figs. 4–7). Prior MEG validation studies report robust lateralization and moderate-to-high concordance with hemodynamic or invasive benchmarks across cohorts, but most rely on rest/fixation or prestimulus baseline contrasts rather than explicit task-matched nonlinguistic controls (e.g., Findlay et al., 2012; Wang et al., 2012). McDonald et al. (2009) is a clear example that includes an explicit false-font control task, whereas Pang et al. (2011) used degraded post-cue stimuli in a way that does not constitute a clean task-matched subtraction because verb generation continued during that interval.

### 4.4. Fixed versus participant/ROI-specific time windows

Fixed post-stimulus bins are common in MEG language mapping, but inter-individual and regional timing can vary by >300 ms, and inferior-frontal versus superior/middle-temporal activity can differ in onset by tens to approximately 100 ms depending on task demands (Bourguignon et al., 2013; Kochi et al., 2023; McDonald et al., 2009; Raghavan et al., 2017; Tanaka et al., 2013). To accommodate this variability, we used an rRI-guided procedure to center a 300-ms window on each participant’s ROI-specific absolute LI peak (Methods; Fig. 6A).

In practice, this participant/ROI-specific peak alignment produced only small and mixed changes in MEG–fMRI agreement relative to a single fixed 300-ms window chosen within 300–1200 ms (Fig. 7, Table 2). For the SD > SYM contrast, agreement differences ranged from slight decrements to modest improvements depending on ROI and LI metric; the largest change was a + 5.6% increase in Frontal agreement for the magnitude-based LI, whereas effects in other ROIs and metrics were smaller and not consistently positive. For SD > Baseline, differences between fixed and participant-specific windows were similarly small and, in several cases, negative (≈−7% to +6% across ROIs and metrics). Thus, although the windowing scheme was motivated by the idea of reducing latency jitter and emphasizing high-rRI epochs, in this dataset it did not yield a robust or systematic improvement in MEG–fMRI concordance and mainly shifted a small number of cases near the decision boundary. However, this approach may be relevant in patients who are outliers in terms of their response latencies due to acquired or developmental differences in brain physiology. Accordingly, we view rRI-guided participant/ROI-specific windowing primarily as an adjunctive or research-oriented analytic option rather than as a required component of a routine clinical pipeline. Its main value may lie in characterizing inter-individual latency variability and in selected outlier cases with atypical response timing, whereas the simpler fixed-window approach appears adequate and more pragmatic for most routine applications in this clinical population.

### 4.5. Threshold-robust laterality indices

Classical LI formulations require choosing a single activation threshold and can be sensitive to that choice and to residual noise, which may introduce hemispheric bias or unstable dominance calls (Seghier, 2008). The weighted-bootstrap LI mitigates this by resampling the beta suppression-magnitude distribution across multiple thresholds and summarizing it with a median estimate and 95% confidence interval that are less affected by outliers, uneven coverage, or small clusters (Wilke and Schmithorst, 2006).

In our data, the three LI formulations produced broadly similar MEG–fMRI concordance (Fig. 5, Table 2). Differences between methods were generally small (on the order of a few percentage points) and mixed across ROIs and contrasts: in some cases, the bootstrap LI was slightly better; in others, magnitude- or vertex-count-based LIs performed as well or slightly better, and no single method consistently dominated. The most consistent pattern across LI methods was that the Lateral ROI tended to yield the highest and most stable MEG–fMRI agreement across both SD > SYM and SD > Baseline contrasts, suggesting that the choice of ROI has a larger impact on concordance than the specific LI variant.

One practical advantage of the bootstrap approach, independent of these small concordance differences, is that it avoids reliance on a single hard threshold and provides both a point estimate and an explicit measure of variability, which may be useful when interpreting borderline cases. In this study, the bootstrap procedure yielded an LI point estimate and a 95% confidence interval for each participant and ROI. These confidence intervals were used only to summarize uncertainty around individual LI estimates; they did not contribute to category assignment or decision rules and are not shown in the group-level analyses.

### 4.6. Relationship to prior MEG literature and invasive standards

In this cohort, the clearest methodological effect was the use of a matched nonlinguistic control (SD > SYM), which substantially increased LI magnitude and MEG–fMRI concordance relative to a prestimulus baseline. By contrast, the other analytic choices we explored, participant/ROI-specific timing and a threshold-robust (bootstrap) LI, had only small, mixed effects on concordance (Sections 4.4–4.5), providing useful negative results about the limits of these optimizations.

Reviews place MEG–IAP concordance broadly at approximately 70 to 95 percent across surgical series (Pirmoradi et al., 2010; Salmelin, 2007; Stufflebeam, 2011; Trébuchon et al., 2020). These summary figures, however, obscure substantial variation in tasks, source analysis methods, case mix, and the size of the IAP-validated subset. ERP- and ERF-based paradigms benchmarked directly against IAP have shown that magnetic source imaging can reproduce IAP language dominance with good accuracy (Doss et al., 2009; Papanicolaou et al., 2004; Pirmoradi et al., 2010), but MEG–IAP overlap samples are often modest and skewed toward strongly left-dominant cases. Within beta-band event-related desynchronization and beamforming pipelines, SAM beamformer studies have reported concordance at the high end of the range (e.g., ~95% in one small series) and close agreement with invasive mapping for dominance and localization (Herfurth et al., 2022; Hirata et al., 2010, 2004). These high concordance values are again based on relatively small overlap groups and may be inflated by sampling variability and selective publication. In other series, particularly when patients with atypical or bilateral IAP outcomes are examined, concordance is clearly lower (e.g., Doss et al., 2009; Herfurth et al., 2022), suggesting that performance across the full clinical spectrum is likely below the upper end of the reported range.

Task design and baseline conditions also vary widely across prior MEG–IAP comparisons. Most MEG–IAP studies have contrasted a single language task with rest, fixation, or a prestimulus baseline, which may not fully subtract nonlinguistic visual, attentional, or motor decision demands (Doss et al., 2009; Hirata et al., 2004; Papanicolaou et al., 2004; Tanaka et al., 2013). Within the MEG–IAP clinical validation literature, explicit task-matched nonlinguistic control conditions are comparatively uncommon. One clear example is McDonald et al. (2009), who used a visual semantic-decision task with a false-font control task. Pang et al. (2011) presented visually degraded stimuli as a post-cue comparator during covert verb generation; because the language task continued during this interval, this design differs from an explicit task-matched nonlinguistic control. Early MEG language mapping studies also used “control” contrasts in a different sense, for example by comparing the distribution of late-window language-related dipole sources with early-window dipole sources elicited by simple sensory stimuli such as tones (Papanicolaou et al., 1998). More broadly, a substantial basic-science MEG literature compares evoked-response magnitudes between language and control conditions within predefined ROIs, even when the goal is not to produce spatial maps of a differential response (Pylkkänen, 2019; Salmelin, 2007). More recently, a healthy-volunteer beta-band MEG study incorporated both active (syllable-repetition) and passive control conditions for a sentence-completion task, and suggested that including an active control may improve the specificity of language-region localization (Protopova et al., 2025). Together, these studies illustrate a range of ways control conditions have been used in MEG language research; the present work builds on this foundation by explicitly mapping a task–control differential response in source space and deriving hemispheric dominance estimates from that differential.

In MEG–IAP series, the limited use and heterogeneous implementation of control conditions make it difficult to isolate how much task subtraction, timing choices, and LI methods contribute to reported concordance differences. In this context, our symbol-matching contrast was designed to more closely match sensory input and overt response format between the linguistic and nonlinguistic conditions, while acknowledging that such matching is necessarily approximate. If the patterns observed in our MEG–fMRI comparison generalize to MEG–IAP, very high concordance estimates from small, selected series may not generalize to larger clinical cohorts that include atypical, bilateral, and lower-SNR cases.

A smaller number of studies have directly compared MEG-derived language laterality with fMRI. These typically report moderate to high concordance, and several suggest stronger agreement for oscillatory or beta-desynchrony measures than for ERF-based approaches (Billingsley-Marshall et al., 2007; Foley et al., 2019; Herfurth et al., 2022; Raghavan et al., 2017). As with the MEG–IAP literature, many MEG–fMRI series rely on relatively small overlap samples and often do not include detailed analyses of where discordance arises or how stable LIs are across regions; most also use a single language task contrasted with rest or fixation and do not combine nonlinguistic controls, individualized timing, and threshold-robust LIs within one framework.

Against this backdrop, a symbol-controlled contrast analyzed with rRI-guided peak windows and a weighted-bootstrap LI yielded 90.5% agreement with fMRI in a relatively large, clinically heterogeneous TLE cohort. The sample size and broad clinical inclusion make this estimate more representative of routine clinical practice than many smaller IAP-validated or MEG–fMRI subsamples. In addition to overall concordance, we examined cross-ROI stability and characterized where discordance arises (e.g., in cases with weaker or regionally heterogeneous LIs), providing a more nuanced picture of performance. Beta-band MEG can approach the upper range of concordance reported against IAP or fMRI when dominance is strong and data quality is adequate, but in large, unselected TLE cohorts, substantial residual variability and occasional modality dissociations are expected and should be recognized explicitly rather than implied to be absent.

### 4.7. Interpreting residual discordance

Discordant cases at the best-performing ROI and LI estimation method (7 of 74) reflected two patterns: cross-hemisphere reversals (*n* = 5) and borderline cases in which one modality was clearly lateralized, and the other was near symmetric (*n* = 2). Beyond these clinically salient cases, we also asked how stable hemispheric dominance was across ROIs within each modality. MEG showed more frequent cross-ROI left↔right reversals than fMRI (27.0% vs. 9.5% of patients) and lower cross-ROI LI correlations (mean off-diagonal $r^{-}=0.45$ for MEG vs. $r^{-}=0.81$ for fMRI), indicating that MEG laterality estimates vary more across ROIs than fMRI LIs within the same participants. At the same time, the primary Lateral composite ROI showed high MEG–fMRI concordance at the group level.

There are at least two non-exclusive interpretations of this pattern. One possibility is that MEG, which is sensitive to fast, transient beta modulations, may pick up genuine regional differences in the timing or strength of language-related oscillatory activity that are harder to detect with fMRI, whose hemodynamic signal integrates activity over longer time windows and across vascular territories. On this view, the seven grossly discordant cases could reflect the extreme tail of a broader tendency for MEG to show more heterogeneous regional lateralization than fMRI.

An alternative and at least equally plausible explanation is that MEG LIs estimated using this source modeling approach, are simply less reliable than fMRI LIs, particularly in smaller ROIs. This would be consistent with the observation that concordance is greatest for the largest ROI (the Lateral composite), where spatial averaging improves signal-to-noise ratio, and with the generally higher internal consistency of fMRI LIs across ROIs. We cannot distinguish between these accounts with the present data, and both mechanisms may contribute. From a practical standpoint, the main implication is that regional MEG LIs should be interpreted cautiously, especially in smaller ROIs or low SNR cases, even when the composite Lateral ROI shows good agreement with fMRI.

An additional, non-exclusive contributor to this pattern is that the MEG and fMRI paradigms were not modality matched: MEG used a visual semantic-decision versus symbol-matching contrast, whereas fMRI used an auditory semantic-decision versus tone-decision contrast. Although both paradigms incorporate sensory modality-specific controls, they likely place somewhat different weights on partially distinct components of the broader language network. The visual MEG task necessarily engages occipital and ventral occipitotemporal regions early and may place relatively greater weight on orthographic/visual-semantic and posterior parietal processes, whereas the auditory fMRI task may place relatively greater weight on superior temporal, temporoparietal, and perisylvian auditory-language pathways. Consequently, some reduction in MEG–fMRI concordance at the level of individual ROIs, and some of the greater cross-ROI variability observed for MEG relative to fMRI, may reflect task-modality differences rather than only measurement noise or true disagreement in hemispheric dominance. This interpretation is also consistent with the stronger performance of the composite Lateral ROI, which should be less sensitive to modality-specific weighting of any single subregion. Because the present dataset did not include modality-matched MEG and fMRI language tasks, we could not quantify this effect directly.

Screening behavioral, clinical, cognitive, seizure-burden, and signal-quality variables revealed no robust FDR-significant predictors of |MEG – fMRI| across ROIs. Temporal lobe epilepsy side (left vs. right seizure onset) did not show any detectable association with discordance rates or with |MEG – fMRI| in any ROI. The only notable association was a mild negative correlation between AED load and MEG LI in the Angular ROI (Fig. S3; Tables S4–S5). For visualization, Fig. S2 highlights only gross discordances (Left↔Right); Neutral↔Left or Neutral↔Right mismatches are not flagged under that rule.

These observations have several practical implications for clinical implementation, although our data do not yet allow us to specify hard decision thresholds. First, we did not identify robust behavioral or clinical predictors of discordance (Section 3.5). Given the known sensitivity of MEG beamformer LIs to data quality and SNR (Taulu and Simola, 2006; Treacher et al., 2021), it seems prudent that clinical MEG pipelines include explicit pre-scan triage and automated quality control steps, such as monitoring source-level SNR, trial loss rates, and residual artifacts, to flag cases in which lateralization estimates are likely to be unstable. Second, conceptually, discordances are most worrisome when they occur in cases with modest LI magnitudes or clear cross-ROI variability, and they are less concerning when both modalities show strong, convergent LIs. Weighted bootstrap LIs with confidence intervals provide threshold-free measures of effect size and uncertainty (Seghier, 2008; Wilke and Schmithorst, 2006), and may therefore help avoid overconfident interpretations when effect sizes are small or when regional LIs disagree. We view these as pragmatic considerations informed by the current pattern of results and prior literature, rather than empirically validated decision rules.

It is also important to recognize the distinct physiological bases of MEG and fMRI. MEG is sensitive to millisecond-scale synchrony in pyramidal-layer currents, whereas fMRI reflects slower vascular responses to net metabolic demand. Neurovascular uncoupling, venous anomalies, or lesion-related perfusion changes can attenuate BOLD signals in otherwise active cortex (Dym et al., 2011; Pillai and Mikulis, 2015), and transient or spatially focal activations may not always translate into robust BOLD signals. Large clinical series in epilepsy surgery candidates show that language fMRI is strongly predictive of post-surgical language outcomes (Bonelli et al., 2012; Gross et al., 2022; Trimmel et al., 2019) and in many respects outperforms the IAP (Binder et al., 2008a; Janecek et al., 2013b; Sabsevitz et al., 2003), but also that a minority of patients have bilateral or equivocal fMRI language maps despite clear language dominance based on other measures or clinical follow-up (Janecek et al., 2013b). These observations highlight that fMRI, like MEG, can be limited or noninformative in some individual cases, particularly in the presence of lesions or vascular abnormalities. Thus, at least some MEG–fMRI mismatches may reflect true modality-specific limitations or dissociations rather than pure measurement error in one modality. Task-modality differences in the present study, with a visual SD > SYM contrast for MEG and an auditory SD > TD contrast for fMRI, may further emphasize different components of the language network, even when overall language dominance is left-lateralized.

### 4.8. Limitations

Several factors temper the generalizability and interpretation of our findings. First, fMRI served as the external benchmark. Although well validated for lateralization, fMRI can be attenuated or displaced by neurovascular uncoupling, venous anatomy, or lesion-related perfusion changes, especially in lesional cortex, so it does not necessarily constitute ground truth (Dym et al., 2011; Janecek et al., 2013a; Pillai and Mikulis, 2015). We did not include Wada/IAP or postoperative language outcomes, which could have provided stronger external validation, since our cohort does not represent drug-resistant temporal lobe epilepsy patients who underwent epilepsy surgery.

Second, this was a single-center study on an Elekta VectorView^™^ system in native English-speaking adults with a wide range of TLE severities. Replication across vendors, magnetic-shielding environments, age groups, linguistic backgrounds (including bilingual and non-native English speakers), and non-TLE pathologies is needed (Pang et al., 2011; Stufflebeam, 2011). In addition, base rates of atypical or neutral language laterality may depend on cohort composition, including handedness distribution and seizure-focus laterality. We intentionally retained the full adult TLE cohort, including left-handed and ambidextrous participants, because our goal was to evaluate MEG–fMRI concordance in a clinically representative presurgical population rather than in a more homogeneous subgroup. Restricting analyses to right-handed participants would have reduced sample size and excluded clinically relevant atypical language laterality patterns. Future larger studies should examine the stability of these findings in subgroups stratified by handedness, age, and seizure-focus laterality.

Third, the MEG and fMRI tasks differed in input modality (visual semantic-decision with a symbol control for MEG; auditory semantic vs. tone decision for fMRI). Both paradigms are validated for lateralization, but the modality difference might differentially weight visual vs. auditory language streams and may contribute to a subset of cross-hemisphere reversals, which we could not quantify here.

Fourth, we did not preregister or predefine QC/SNR thresholds for inclusion or exclusion. Standardizing pre-scan triage (e.g., head motion guidance, interference control) and adopting prospectively defined SNR cutoffs would likely reduce variance across centers. Our DICS/beamformer pipeline also carries well-known caveats (spatial leakage, depth/orientation bias), and although tSSS and MEGnet mitigate artifacts, residual contamination can persist.

Fifth, some analysis choices involve tunable parameters. We used a symmetry-safe suprathreshold for magnitude/count LIs (*T* = 0.5 × max s (v)), a ± 10 “neutral zone” for categorical labels, and rRI-guided windows defined within 300–1200 ms using θ = 0.05 × P95(ΔrRI). While these choices are principled and produced stable results, they are not unique. Multicenter work should predefine and harmonize thresholds, including the neutral zone for LI categorization and any criteria for using confidence intervals in decision-making, and should examine sensitivity to atlas, window, and parameter settings (Seghier, 2008; Wilke and Schmithorst, 2006).

Sixth, LI estimates depend on ROI definition and parcellation. We grouped HCP-MMP parcels into angular, frontal, temporal, and composite lateral ROIs. Alternative atlases, area-weighted ROIs, or different composite schemes could shift absolute LI values and should be explored in future work. Although the beamformer maps showed sustained task-related effects in ventral sensorimotor/articulatory, insular, and supramarginal-adjacent regions, these areas were not added post hoc to the LI masks. We cannot exclude the possibility that alternative or expanded ROI schemes would shift absolute LI values or MEG–fMRI concordance, and this should be examined in future work.

Seventh, in the presence of highly correlated neural activity (which is possible during broad engagement of language areas), DICS beamforming can potentially attenuate source magnitudes and thereby introduce localization biases in the source maps, spurious activation patterns, or distortion of time courses. The degree to which this may be a factor could be assessed in future studies using modified methods such as the dual-core beamformer (Diwakar et al., 2011), or estimation of beta power using inverse solutions, although the latter may be more susceptible to noise.

Finally, the design is cross-sectional, and the number of discordant cases was small (*n* = 7). As a result, multivariable modeling had limited power, and we cannot address prognostic value. Prospective studies relating MEG LI (and CI strength) to postoperative naming/memory change are needed to establish predictive utility (Busch et al., 2018; Hamberger et al., 2003).

### 4.9. Clinical implications and future directions

An approximately 15-minute MEG acquisition using a symbol-controlled contrast yielded approximately 90% categorical agreement with fMRI-based language laterality classifications in this TLE cohort. This suggests that relatively simple protocol choices—particularly the use of a nonlinguistic control task and a composite lateral ROI—may improve the reliability of MEG-derived laterality estimates. In contrast, participant- and ROI-specific timing produced only small and mixed effects on concordance in this dataset and does not appear necessary as a default step for routine clinical deployment. Rather, it may be most useful as an adjunctive analysis for atypical or borderline cases, or for characterizing inter-individual variability in response timing. The bootstrap LI also provides confidence intervals, which may help flag borderline cases for closer review, although we did not formally evaluate CI-based decision rules in this study.

The current symbol-matching control reduces explicit reading demands compared with the word-based task, although it remains cognitively demanding. In principle, simpler variants (e.g., with a single visual target) could be developed for patients with more limited reading skills or cognitive impairment, but these were not tested here. MEG is also feasible in many patients who cannot undergo MRI, for example, because of implanted devices, severe claustrophobia, or a high risk of neurovascular uncoupling that may reduce the interpretability of BOLD signals (Bagić et al., 2011; Burgess et al., 2011; Pillai and Mikulis, 2015). In clinical practice, MEG is best viewed as a complementary modality that can act as a second-line adjudicator when fMRI results are equivocal and may help reduce IAP referrals to a smaller subset of patients in whom noninvasive methods remain discordant.

A multicenter, preregistered study that spans MEG vendors, shielded-room designs, and linguistic communities is a logical next step. Such a study should implement unified quality control and SNR metrics and clearly specified dominance rules, for example, a ± 10% neutral zone and consistent label criteria. On the acquisition side, higher-density arrays, active shielding, and improved head tracking may increase data quality in challenging patients (Baillet, 2017; Stufflebeam, 2011). Analytically, adaptive beamforming, area-weighted ROIs, and connectomic integration of LIs may help identify secondary hubs at risk. Finally, prospective outcome studies are needed to test whether MEG LI and its associated uncertainty measures predict postoperative naming and memory change. If these relationships are confirmed, MEG could serve as a screening tool or gatekeeper for targeted invasive testing within presurgical language evaluation, rather than as a complete replacement for Wada (Hirata et al., 2004; Janecek et al., 2013a; Tanaka et al., 2013).

### 4.10. Conclusion

A brief semantic-decision task, analyzed using a task-matched nonlinguistic symbol-matching control, yielded beta-band MEG laterality estimates that showed approximately 90% concordance with fMRI in a large, clinically heterogeneous temporal lobe epilepsy cohort. The resulting pattern captures a physiologically plausible left-hemisphere lateralization cascade from frontal to temporal–parietal regions.

Taken together, these results provide a practical template for a time-resolved MEG pipeline for presurgical language mapping. The approach is brief to acquire and readily integrated alongside clinical fMRI. In combination with existing invasive standards, such a pipeline may help reduce reliance on IAP testing and extend reliable language mapping to patients for whom standard fMRI protocols are less suitable, although these broader applications will require direct validation in future MEG–IAP and MEG–stimulation studies.

## Supplementary Material

MMC1

MMC2

Supplementary material associated with this article can be found, in the online version, at doi:10.1016/j.neuroimage.2026.122051.

## Figures and Tables

**Table 1:** Demographic and clinical characteristics of the study cohort. Values are n (%) or mean ± SD unless otherwise noted. Age at first seizure is reported as median (IQR), and AED load as median (range). TLE = temporal lobe epilepsy; IQR = interquartile range; AED = antiepileptic drug; WASI-II = Wechsler Abbreviated Scale of Intelligence, Second Edition; FSIQ-2 = two-subtest estimated Full Scale IQ.

Characteristic	Value

**Sample size**	74 adults with drug-resistant TLE
**Sex**	26 men (35%) • 48 women (65%)
**Age**	39.1 ± 11.9 y (range 19–60 y)
**Handedness**	Right 59 (79.7%) • Left 9 (12.2%) • Ambidextrous 4 (5.4%) • Missing 2 (2.7%)
**Native language**	100% native English speakers
**Seizure focus**	Left TLE 51 (68.9%) • Right TLE 15 (20.3%) • Bilateral 4 (5.4%) • Uncertain 2 (2.7%) • Missing 2 (2.7%)
**Age at first seizure**	Median 15 y (IQR 12–25 y)
**AED load**	Median 2 drugs (range 0–5)
**Estimated FSIQ-2**	99 ± 13 (range 72–140)

Estimated FSIQ-2 was derived from the WASI-II Vocabulary and Block Design subtests using age-normed scores and the corresponding two-subtest conversion in the WASI-II manual.

**Table 2:** MEG–fMRI language laterality concordance in adults with temporal lobe epilepsy (*N* = 74). Values are the percent of participants whose MEG LI category (left/right/symmetric) matched fMRI. Results are shown for three LI metrics (Source Magnitude, Vertex Counting, Weighted-Bootstrap) across four HCP- MMP ROI sets (Angular, Frontal, Temporal, Lateral). For each metric × ROI, two contrasts are reported—SD > Baseline (semantic-decision vs. prestimulus baseline) and SD > SYM (semantic-decision vs. symbol-matching)—each computed in (i) a fixed 300-ms cohort window selected within 0.30–1.20 s, and (ii) a patient-specific 300-ms window selected within 0.30–1.20 s using a regional response index (rRI)-guided bound. Bold indicates the highest concordance (90.5%).

Metric	ROI	SD vs. Base		SD vs. SYM	
		Fixed interval	Patient-Specific	Fixed interval	Patient-Specific

Magnitude	Angular	69	66.2	81.9	80.5
	Frontal	71.8	66.2	80.5	86.1
	Temporal	63.3	66.2	84.7	84.7
	Lateral	71.8	73.2	88.8	88.8
Vertex Counting	Angular	69	64.8	81.9	83.3
	Frontal	71.8	66.2	83.3	80.5
	Temporal	63.3	69	84.7	81.9
	Lateral	71.8	71.8	86.1	87.5
Bootstrap	Angular	67.6	63.3	83.3	81.9
	Frontal	71.8	64.7	84.7	83.3
	Temporal	63.3	63.3	83.3	86.1
	Lateral	67.6	70.4	87.5	90.5

Percentages represent the proportion of participants whose MEG LI direction (left, right or bilateral) agreed with their fMRI LI direction.

## Data Availability

Analyses used MATLAB R2019a, FieldTrip (https://www.fieldtriptoolbox.org), and Brainstorm (https://neuroimage.usc.edu/brainstorm); anatomical processing used FreeSurfer (https://surfer.nmr.mgh.harvard.edu). MEGnet is available at https://github.com/nih-megcore/MegNET_2020. The Brainstorm plug-in for source magnitude, counting, and weighted-bootstrap LIs is at https://github.com/vyoussofzadeh/meg-laterality-for-Brainstorm. ECP data are publicly available at https://osf.io/exbt4/.
