# Supplementary material for: Determining hemispheric language dominance from MEG beta-power modulations: Concordance with fMRI
Source: Neuroimage. Author manuscript; Available in PMC 2026 Jul 17. (PMC13373884; doi:10.1016/j.neuroimage.2026.122051)
Supplement: MMC1 [file NIHMS2187318-supplement-MMC1.docx]

Supp. figs


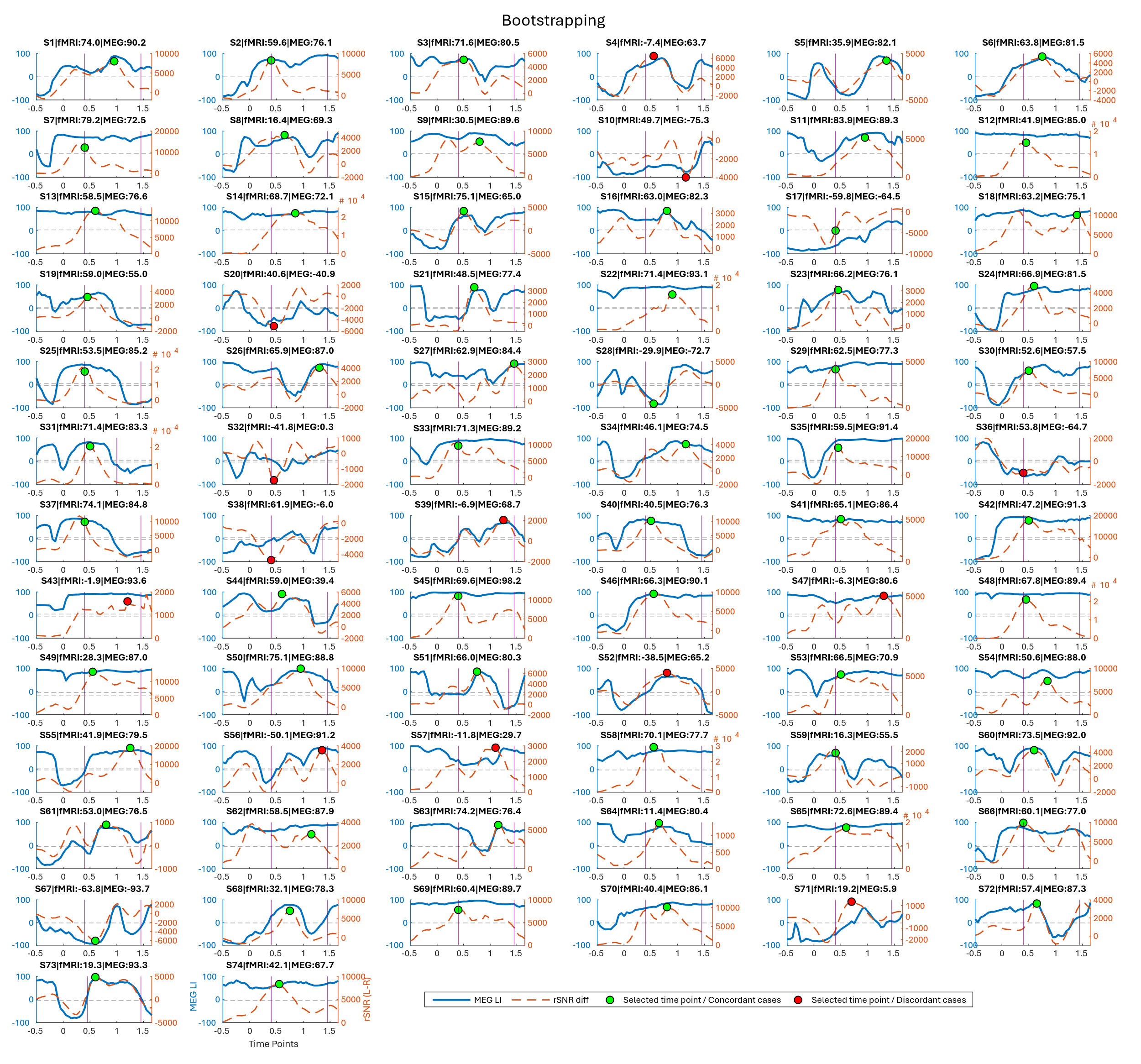


**Figure S1 | Subject-specific laterality trajectories and regional Response Index (rRI) used to define peak windows**. Each mini-panel shows one participant’s Lateral composite bootstrap LI (blue) and the decision signal ΔrRI(t) = |rRI_L(t) − rRI_R(t)| (orange, dashed), computed in 300-ms sliding windows stepped every 10 ms from −0.3 to 1.8 s relative to stimulus onset (vertical gray line at 0 s). High-rRI epochs were defined by θ = 0.05 × P95(ΔrRI) within the 0.30–1.20 s search interval; the 300-ms analysis window was then centered on the |LI(t)| maximum inside those epochs (ties broken by higher ΔrRI, then earlier time). Green circles mark the selected center time in concordant MEG–fMRI cases (e.g., “S19 | fMRI = +59, MEG = +55”), whereas red circles mark the selected center time in discordant cases (e.g., “S10 | fMRI = +49.7, MEG = -75.3”). Titles list participant ID, fMRI LI, and the MEG LI at the selected peak. The plots illustrate (i) heterogeneity of individual LI waveforms, (ii) inter-subject spread of peak latencies (~0.35–1.20 s), and (iii) the positive relation between ΔrRI and LI amplitude, motivating rRI-guided peak selection.


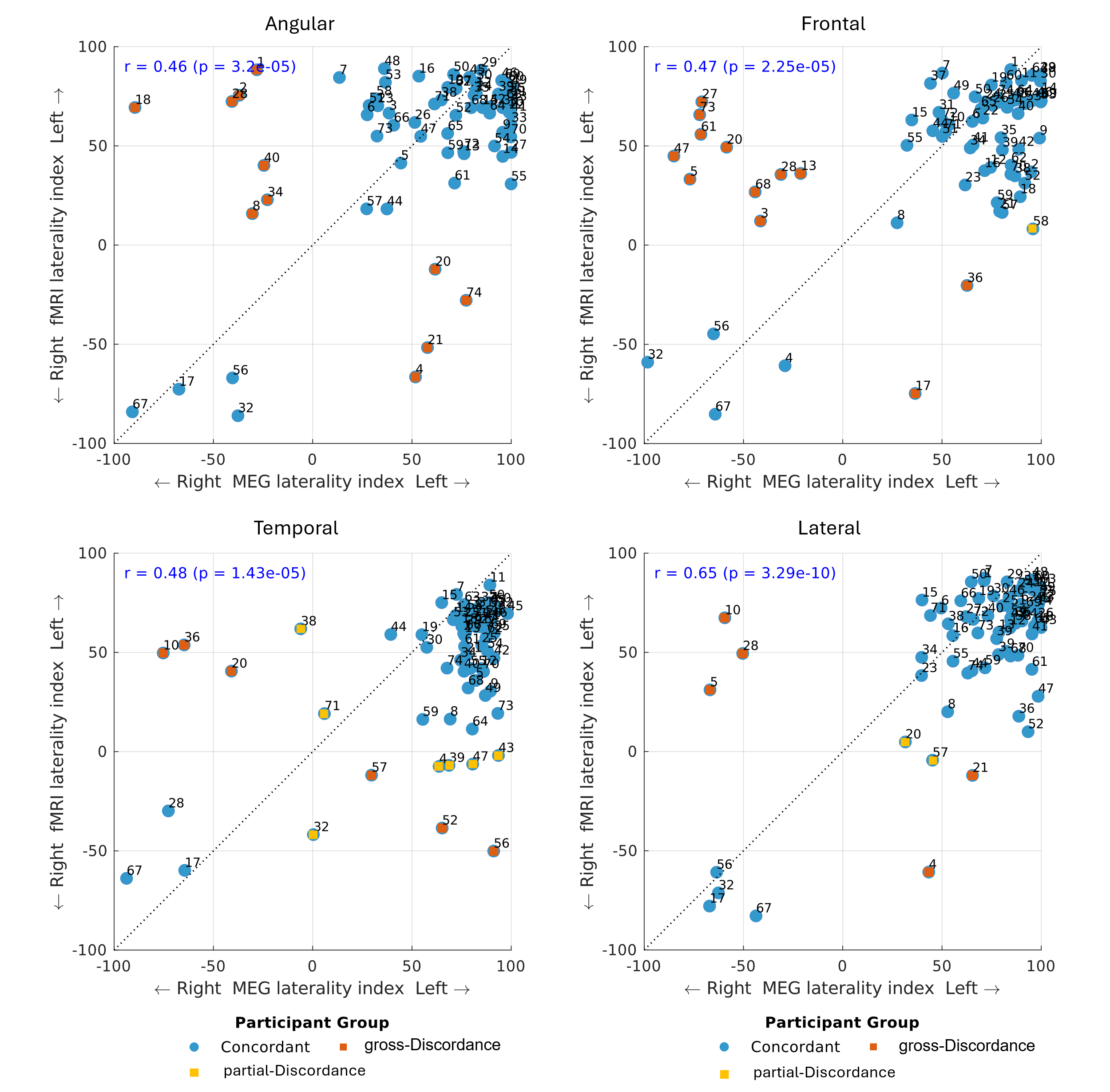


**Figure S2 | Cross-modal agreement between MEG and fMRI laterality indices by ROI.** Per-participant scatterplots (n = 74) for Angular, Frontal, Temporal, and Lateral networks. Axes show MEG LI (x) vs fMRI LI (y), oriented −100 = right and +100 = left; the dotted line is identity. MEG LIs are Bootstrap point estimates taken from rRI-guided, participant-/ROI-specific 300-ms peak windows; fMRI LIs are weighted-bootstrap estimates from the matched ROI set. Panel insets report Pearson r (two-sided p). Color coding: blue = concordant categorical labels (left/right/symmetric), orange = gross discordance (left↔right reversal with opposite-signed LIs), yellow = partial discordance (one modality clearly lateralized while the other lies in the neutral zone, |LI| ≤ 10).

**
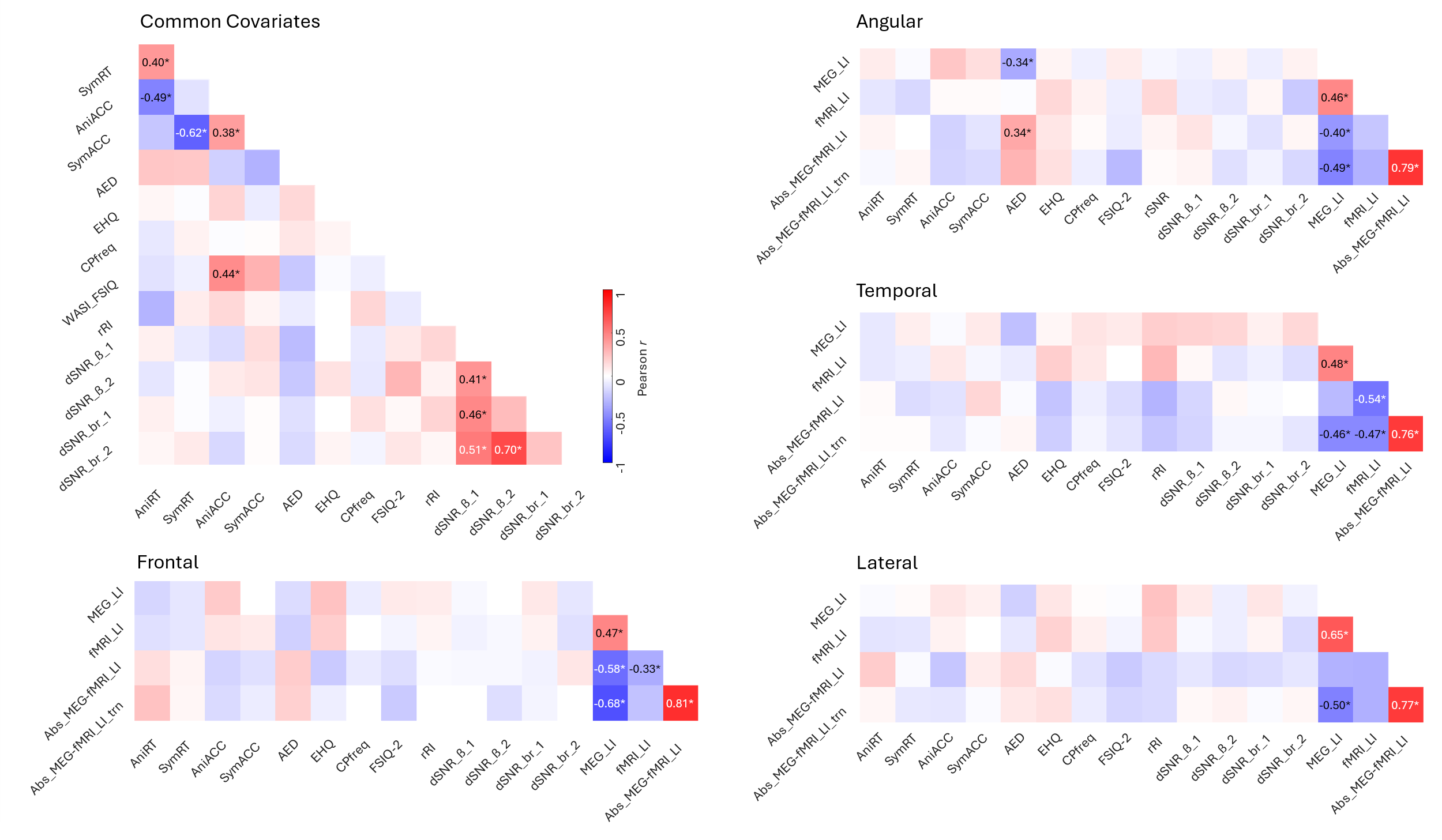
**

**Figure S3 | Correlation structure of behavioral, clinical, cognitive, and data-quality variables with MEG and fMRI laterality.** Left: Lower-triangle Pearson correlation matrix (r) for 14 covariates across 74 participants: Symbol-task reaction time (Sym RT), Animal-task accuracy (Anim ACC), Symbol-task accuracy (Sym ACC), number of antiepileptic drugs (AED), Edinburgh Handedness Quotient (EHQ), interictal spike frequency on clinical EEG (CP freq), Full-Scale IQ (WASI_FSIQ), source-level relative SNR (rRI), and trial-level SNR estimates in narrow-band (Tri SNR 0.1, Tri SNR 0.2) and broadband (Tri SNR Broad 1, Tri SNR Broad 2) windows. Warm colors indicate positive correlations; cool colors indicate negative correlations; cells with FDR-corrected p < 0.05 are annotated with r. Right: For each HCP-MMP ROI (Angular, Frontal, Temporal, Lateral composite), heatmaps relate these covariates to four outcomes: MEG LI, fMRI LI, the absolute MEG–fMRI LI difference |MEG − fMRI|, and the absolute ternary classification difference |MEG_trn − fMRI_trn|. Significant correlations after FDR correction are labeled. Across ROIs, patterns were sparse beyond the internal LI relationships (MEG LI correlating with fMRI LI; |MEG − fMRI| decreasing as either LI strengthened). The only notable covariate–LI association was a mild negative correlation between AED load and MEG LI in the Angular ROI; no other behavioral, clinical, cognitive, or SNR covariates showed robust FDR-significant associations.


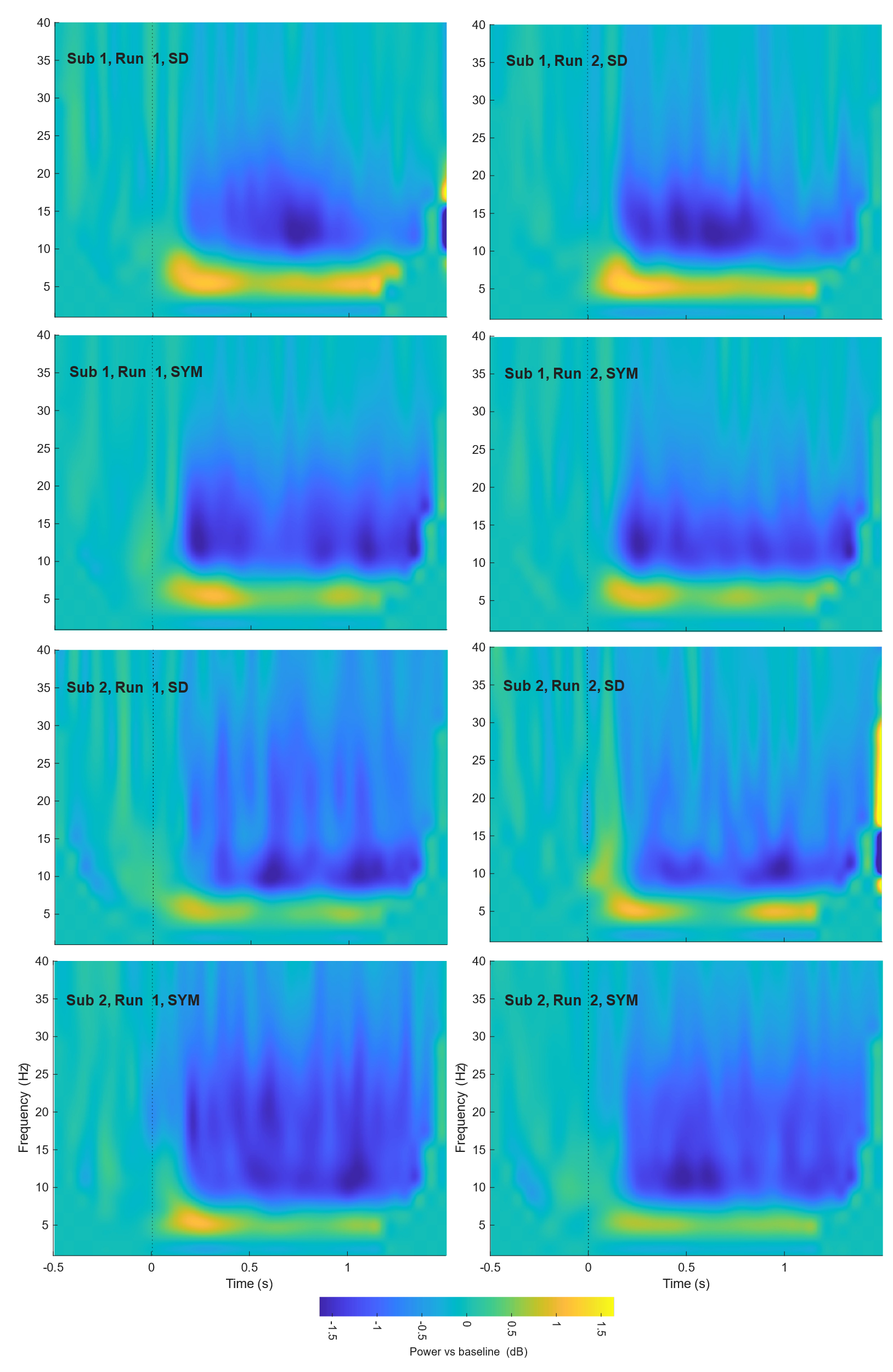


**Figure S4 | Illustrative single-subject time-frequency representations for the semantic-decision (SD) and symbol-matching (SYM) tasks**. Sensor-level power relative to baseline is shown for two illustrative participants, separately for SD and SYM and for each of the two runs. Warm colors indicate power increases and cool colors indicate power decreases relative to baseline. The vertical dashed line marks stimulus onset (0 s). Across both example participants, SD and SYM show broadly similar early visual/perceptual responses, while both conditions also exhibit evolving low-beta suppression after stimulus onset. These examples are provided to illustrate within-subject task dynamics; they are not intended as a group-level statistical summary.
