# Supplementary material for: Determining hemispheric language dominance from MEG beta-power modulations: Concordance with fMRI
Source: Neuroimage. Author manuscript; Available in PMC 2026 Jul 17. (PMC13373884; doi:10.1016/j.neuroimage.2026.122051)
Supplement: MMC2 [file NIHMS2187318-supplement-MMC2.docx]

Supp. Table

Table S1 | Stimuli used in the Semantic-Decision task and their category assignments. For each of the 128 animal names, the table indicates whether it was classified as USED BY PEOPLE and/or FOUND IN THE UNITED STATES. A “yes” response was correct only if a stimulus satisfied both criteria. Names are shown in uppercase to match on-screen presentation. Y = yes; N = no.

| No. | STIMULUS | USED BY PEOPLE | FOUND IN THE UNITED STATES | CORRECT SD RESPONSE |
| --- | --- | --- | --- | --- |
| 1 | LEOPARD | N | N | N |
| 2 | FOX | YES | YES | YES |
| 3 | ELEPHANT | N | N | N |
| 4 | CLAM | YES | YES | YES |
| 5 | CATERPILLAR | N | YES | N |
| 6 | SOW | YES | YES | YES |
| 7 | HOG | YES | YES | YES |
| 8 | BEAR | N | YES | N |
| 9 | COYOTE | N | YES | N |
| 10 | MOOSE | N | YES | N |
| 11 | MOUSE | N | YES | N |
| 12 | SKUNK | N | YES | N |
| 13 | RHINOCEROS | N | N | N |
| 14 | SHRIMP | YES | YES | YES |
| 15 | JAGUAR | N | N | N |
| 16 | CALF | YES | YES | YES |
| 17 | CAMEL | YES | N | N |
| 18 | OYSTER | YES | YES | YES |
| 19 | FLAMINGO | N | YES | N |
| 20 | RABBIT | YES | YES | YES |
| 21 | MOTH | N | YES | N |
| 22 | PENGUIN | N | N | N |
| 23 | GERBIL | YES | YES | YES |
| 24 | BOAR | N | YES | N |
| 25 | SHREW | N | YES | N |
| 26 | SQUIRREL | N | YES | N |
| 27 | PIGEON | N | YES | N |
| 28 | PONY | YES | YES | YES |
| 29 | TURKEY | YES | YES | YES |
| 30 | COUGAR | N | YES | N |
| 31 | PYTHON | N | N | N |
| 32 | STORK | N | YES | N |
| 33 | RAVEN | N | N | N |
| 34 | FERRET | N | N | N |
| 35 | SNAKE | N | N | N |
| 36 | MINK | YES | YES | YES |
| 37 | SEAL | N | N | N |
| 38 | GOLDFINCH | N | N | N |
| 39 | FLY | N | N | N |
| 40 | HALIBUT | N | N | N |
| 41 | COBRA | N | N | N |
| 42 | WREN | N | N | N |
| 43 | PHEASANT | N | N | N |
| 44 | EMU | N | N | N |
| 45 | STEER | YES | YES | YES |
| 46 | WOLF | N | N | N |
| 47 | SPIDER | N | YES | N |
| 48 | PIG | YES | YES | YES |
| 49 | KOALA | N | N | N |
| 50 | GROUSE | N | N | N |
| 51 | OTTER | N | N | N |
| 52 | HERON | N | N | N |
| 53 | BABOON | N | N | N |
| 54 | RATTLESNAKE | N | N | N |
| 55 | DEER | YES | YES | YES |
| 56 | CANARY | N | N | N |
| 57 | ALLIGATOR | N | N | N |
| 58 | SEAHORSE | N | N | N |
| 59 | MOLE | N | N | N |
| 60 | OX | N | N | N |
| 61 | OCTOPUS | N | N | N |
| 62 | PERCH | YES | YES | YES |
| 63 | WORM | N | N | N |
| 64 | BAT | N | N | N |
| 65 | EEL | N | N | N |
| 66 | HIPPOPOTAMUS | N | N | N |
| 67 | SALMON | YES | YES | YES |
| 68 | CRANE | N | N | N |
| 69 | BULL | N | N | N |
| 70 | MOSQUITO | N | N | N |
| 71 | MULE | YES | YES | YES |
| 72 | BLUEGILL | N | N | N |
| 73 | GOAT | YES | YES | YES |
| 74 | KITTEN | YES | YES | YES |
| 75 | WOLVERINE | N | N | N |
| 76 | MAGPIE | N | N | N |
| 77 | STARFISH | N | N | N |
| 78 | SEAGULL | N | N | N |
| 79 | RACCOON | N | N | N |
| 80 | ROOSTER | N | N | N |
| 81 | HEDGEHOG | N | N | N |
| 82 | PANDA | N | N | N |
| 83 | PEACOCK | N | N | N |
| 84 | BASS | YES | YES | YES |
| 85 | PLATYPUS | N | N | N |
| 86 | HUMMINGBIRD | N | N | N |
| 87 | AARDVARK | N | N | N |
| 88 | TUNA | YES | YES | YES |
| 89 | PIRANHA | N | N | N |
| 90 | SNAIL | N | N | N |
| 91 | WOODPECKER | N | N | N |
| 92 | WALLEYE | N | N | N |
| 93 | SCORPION | N | N | N |
| 94 | CHINCHILLA | N | N | N |
| 95 | SHEEP | YES | YES | YES |
| 96 | MALLARD | N | N | N |
| 97 | LARK | N | N | N |
| 98 | ANTELOPE | N | N | N |
| 99 | EAGLE | N | N | N |
| 100 | STARLING | N | N | N |
| 101 | MINNOW | N | N | N |
| 102 | GOOSE | YES | YES | YES |
| 103 | HERRING | N | N | N |
| 104 | JELLYFISH | N | N | N |
| 105 | YAK | N | N | N |
| 106 | NEWT | N | N | N |
| 107 | BUFFALO | N | N | N |
| 108 | TOAD | N | N | N |
| 109 | LAMB | YES | YES | YES |
| 110 | WHALE | N | N | N |
| 111 | FROG | N | N | N |
| 112 | FALCON | N | N | N |
| 113 | BARRACUDA | N | N | N |
| 114 | DOVE | N | N | N |
| 115 | LEMUR | N | N | N |
| 116 | MANATEE | N | N | N |
| 117 | HORSE | YES | YES | YES |
| 118 | LOCUST | N | N | N |
| 119 | QUAIL | N | N | N |
| 120 | WEASEL | N | N | N |
| 121 | SHARK | N | N | N |
| 122 | HARE | N | N | N |
| 123 | CENTIPEDE | N | N | N |
| 124 | CHICKEN | YES | YES | YES |
| 125 | CARIBOU | N | N | N |
| 126 | BEE | N | N | N |
| 127 | MONGOOSE | N | N | N |
| 128 | PORPOISE | N | N | N |

**Table S2 | Cortical regions of interest (ROIs) grouped into four language-relevant networks from the HCP-MMP1.0 atlas (Glasser et al., 2016).** Labels are shown without hemisphere prefix; left/right homologues were used in analyses.

| ROI Group | ROI Labels |
| --- | --- |
| Angular | V7, IPS1, TPOJ3, PGp, IP1, PGi, PGs, V6A |
| Frontal | 10d, 10r, 10v, 11ROI, 13ROI, 23d, 24dd, 24dv, 33pr, 44, 45, 46, 47ROI, 47m, 47s, 55b, 6a, 6ma, 8Ad, 8Av, 8BROI, 8BM, 8C, 9-46d, 9a, 9m, 9p, AVI, FOP4, FOP5, IFJa, IFJp, IFSa, IFSp, SCEF, SFROI, a10p, a32pr, a47r, a9-46v, d32, i6-8, p10p, p32pr, p47r, p9-46v, s32, s6-8 |
| Temporal | FFC, EC, PreS, H, PeEc, STGa, A5, PHA1, PHA3, STSda, STSdp, STSvp, TGd, TE1a, TE1p, TE2a, TF, TE2p, PHT, PH, PHA2, VVC, TGv, STSva, TE1m |
| Lateral | PEF, V7, IPS1, 7PROI, MIP, 47m, 8Av, 8C, 44, 45, 47ROI, a47r, IFJa, IFJp, IFSp, IFSa, p9-46v, 13ROI, 47s, i6-8, AVI, AAIC, STGa, A5, STSda, STSdp, STSvp, TGd, TE1a, TE1p, TE2a, TE2p, PHT, PH, TPOJ3, PGp, IP1, IP0, PGi, PGs, FOP5, p47r, TGv, STSva, TE1m |

**Table S3 | Summary of Behavioral, Clinical, Cognitive, and Signal-Quality Variables Used in Discordance Analysis**. Seventeen continuous variables were included in correlation and regression analyses to identify predictors of MEG–fMRI laterality mismatch. Fourteen of these were discretized for Fisher’s exact tests in Tables S4–S5. Variables span task performance (reaction time and accuracy for semantic-decision and symbol-matching), epilepsy burden (e.g., antiepileptic drug count, spike rate), cognitive ability (WASI-II two-subtest estimated Full-Scale IQ [FSIQ-2]), MEG signal quality and artifact suppression (beta and broadband), and language laterality indices. MEG–fMRI discordance was quantified as both absolute LI difference and absolute ternary difference (|MEG_trn – fMRI_trn|). Abbreviations: AED = antiepileptic drugs; SNR = signal-to-noise ratio; LI = laterality index.

| Variable (Short) | Full Variable Name | Domain | Description |
| --- | --- | --- | --- |
| AniRT | Animal_RT | Task performance | Mean reaction time (s) in semantic-decision task |
| SymRT | Symbol_RT | Task performance | Mean reaction time (s) in symbol-matching task |
| AniACC | Animal_ACC | Task performance | Accuracy (%) in semantic-decision task |
| SymACC | Symbol_ACC | Task performance | Accuracy (%) in symbol-matching task |
| AED | AEDCount | Epilepsy burden | Number of concurrent antiepileptic drugs |
| EHQ | EHQ | Handedness | Edinburgh Handedness Quotient |
| CPfreq | CP_freq | Epilepsy burden | Complex-partial seizure frequency (from clinical EEG) |
| FSIQ-2 | NP1WASI_FSIQ | Cognition | WASI-II two-subtest estimated Full-Scale IQ (FSIQ-2) |
| rRI | rRI | MEG signal quality | Stronger hemispheric sum of rectified DICS beta-suppression within the ROI (max{L,R}) |
| dSNR_Beta_1 | nSNR_Beta_tSSS_vs_Raw | Artifact suppression | SNR gain (beta-band) from raw to tSSS pipeline |
| dSNR_Beta_2 | nSNR_Beta_MEGnet_vs_tSSS | Artifact suppression | SNR gain (beta-band) from tSSS to MEGnet-cleaned data |
| dSNR_br_1 | nSNR_Broad_tSSS_vs_Raw | Artifact suppression | SNR gain (broadband) from raw to tSSS |
| dSNR_br_2 | nSNR_Broad_MEGnet_vs_tSSS | Artifact suppression | SNR gain (broadband) from tSSS to MEGnet-cleaned data |
| MEG_LI | optMEG_LI | Laterality | MEG laterality index (participant-specific, bootstrap-based) |
| fMRI_LI | fMRI_LI | Laterality | fMRI laterality index (bootstrap-based) |
| Abs_MEG-fMRI_LI | MEG_fMRI_abs_diff | Discordance | Absolute difference between MEG and fMRI LIs (\|MEG – fMRI\|) |
| Abs_MEG-fMRI_LItrn | MEG_fMRI_abs_diff_trn | Discordance | Absolute difference between ternary dominance codes (\|MEG_trn – fMRI_trn\|) |

**Table S4 | Fisher’s exact-test screening (2 × 2) of behavioral, clinical, and data-quality covariates versus MEG–fMRI laterality concordance, by HCP-MMP ROI**. Reported are Benjamini–Hochberg–adjusted q-values (qVal). “Counts” are raw cell totals in the form [a, b; c, d], where a = concordant in category 1, b = discordant in category 1, c = concordant in category 2, d = discordant in category 2. No comparison survived FDR control (q < 0.05).

| Measure | Comparison | ROI | qVal | Counts |
| --- | --- | --- | --- | --- |
| Symbol ACC | {Low} vs {High,Mid} | Angular | 0.59 | [1,10; 0, 63] |
| — | — | Frontal | 1 | [0,12; 1, 61] |
| — | — | Lateral | 1 | [0,5; 1, 68] |
| — | — | Temporal | 1 | [0,6; 1, 67] |
| Animal ACC | — | Angular | 0.52 | [1,10; 2, 61] |
| — | — | Frontal | 0.27 | [2,10; 1, 61] |
| — | — | Lateral | 0.38 | [1,4; 2, 67] |
| — | — | Temporal | 1 | [0,6; 3, 65] |
| Animal RT | {Moderate,Slow} vs {Fast} | Angular | 0.79 | [11,0; 55, 8] |
| — | — | Frontal | 0.79 | [12,0; 54, 8] |
| — | — | Lateral | 1 | [5,0; 61, 8] |
| — | — | Temporal | 0.79 | [5,1; 61, 7] |
| Symbol RT | — | Angular | 1 | [10,1; 60, 3] |
| — | — | Frontal | 1 | [12,0; 58, 4] |
| — | — | Lateral | 1 | [5,0; 65, 4] |
| — | — | Temporal | 1 | [6,0; 64, 4] |
| EHQ | {Left} vs {Right} | Angular | 0.77 | [0,11; 6, 53] |
| — | — | Frontal | 0.77 | [2,10; 4, 54] |
| — | — | Lateral | 1 | [0,5; 6, 59] |
| — | — | Temporal | 0.77 | [1,5; 5, 59] |
| TLE side | {Right} vs {Left} | Angular | 1 | [2,7; 14, 45] |
| — | — | Frontal | 0.92 | [4,6; 12, 46] |
| — | — | Lateral | 1 | [0,4; 16, 48] |
| — | — | Temporal | 1 | [1,4; 15, 48] |
| AED | {1,2} vs {3plus} | Angular | 0.85 | [7,4; 47, 14] |
| — | — | Frontal | 0.85 | [8,4; 46, 14] |
| — | — | Lateral | 1 | [4,1; 50, 17] |
| — | — | Temporal | 0.85 | [4,2; 50, 16] |
| LTGTC | {0,1-5,6-20} vs {21plus} | Angular | 0.47 | [8,3; 53, 8] |
| — | — | Frontal | 0.45 | [7,3; 54, 8] |
| — | — | Lateral | 0.57 | [4,1; 57, 10] |
| — | — | Temporal | 0.45 | [4,2; 57, 9] |
| SG | {1to2,0} vs {3plus} | Angular | 1 | [11,0; 61, 2] |
| — | — | Frontal | 1 | [12,0; 60, 2] |
| — | — | Lateral | 1 | [5,0; 67, 2] |
| — | — | Temporal | 1 | [6,0; 66, 2] |
| CP freq | {1to5,0} vs {11plus,6to10} | Angular | 1 | [11,0; 58, 5] |
| — | — | Frontal | 0.71 | [10,2; 59, 3] |
| — | — | Lateral | 1 | [5,0; 64, 5] |
| — | — | Temporal | 0.71 | [5,1; 64, 4] |
| tSSS-2cat - Broad | {Low} vs {High} | Angular | 1 | [6,5; 28, 35] |
| — | — | Frontal | 1 | [6,6; 28, 34] |
| — | — | Lateral | 1 | [2,3; 32, 37] |
| — | — | Temporal | 1 | [4,2; 30, 38] |
| megnet-2cat - Broad | — | Angular | 0.43 | [8,3; 28, 35] |
| — | — | Frontal | 0.67 | [7,5; 29, 33] |
| — | — | Lateral | 0.67 | [3,2; 33, 36] |
| — | — | Temporal | 0.67 | [4,2; 32, 36] |
| tSSS-2cat - Beta | — | Angular | 1 | [7,4; 30, 33] |
| — | — | Frontal | 1 | [7,5; 30, 32] |
| — | — | Lateral | 1 | [1,4; 36, 33] |
| — | — | Temporal | 1 | [3,3; 34, 34] |
| megnet-2cat - Beta | — | Angular | 1 | [5,6; 29, 34] |
| — | — | Frontal | 0.7 | [4,8; 30, 32] |
| — | — | Lateral | 0.7 | [1,4; 33, 36] |
| — | — | Temporal | 0.35 | [5,1; 29, 39] |

**Table S5 | Freeman–Halton (2 × 3) extensions of Fisher’s exact test for three-level covariates versus MEG–fMRI laterality concordance, by HCP-MMP ROI.** Reported are Benjamini–Hochberg–adjusted q-values (qVal). “Counts” are [D₁, D₂, D₃; C₁, C₂, C₃], where D = discordant and C = concordant in the order of class levels shown. No test survived FDR control (q < 0.05); the closest trend was Animal-task accuracy in the Angular ROI (q = 0.08).

| Measure | Comparison; 2x3 (Discord vs Concord); | ROI | qVal | Counts |
| --- | --- | --- | --- | --- |
| Symbol ACC (2by3) | Classes = {Low,Mid,High} | Angular | 0.62 | [1,0,2;17,8,46] |
| — | — | Frontal | 1 | [0,1,3;16,9,45] |
| — | — | Lateral | 1 | [0,1,1;18,4,50] |
| — | — | Temporal | 1 | [0,1,1;18,5,49] |
| Animal ACC (2by3) | — | **Angular** | **0.08** | **[1,2,6;14,4,47]** |
| — | — | Frontal | 0.14 | [2,1,3;17,7,44] |
| — | **—** | **Lateral** | **0.08** | **[1,2,2;18,2,49]** |
| — | — | Temporal | 0.74 | [0,3,2;18,4,47] |
| Animal RT (2by3) | Classes = {Fast,Moderate,Slow} | Angular | 0.88 | [0,8,11;54,0,1] |
| — | — | Frontal | 0.88 | [0,8,12;53,0,1] |
| — | — | Lateral | 1 | [0,8,5;60,0,1] |
| — | — | Temporal | 0.88 | [1,7,5;60,0,1] |
| Symbol RT (2by3) | — | Angular | 1 | [1,3,8;53,2,7] |
| — | — | Frontal | 1 | [0,4,11;50,1,8] |
| — | — | Lateral | 1 | [0,4,5;56,0,9] |
| — | — | Temporal | 1 | [0,4,5;56,1,8] |
| TLE side (2by3) | Classes = {Left,Right,Bilateral} | Angular | 0.34 | [7,45,2;14,2,2] |
| — | — | Frontal | 0.34 | [6,46,4;12,1,3] |
| — | — | Lateral | 0.34 | [4,48,0;16,1,3] |
| — | — | Temporal | 0.40 | [4,48,1;15,1,3] |
| EHQ (2by3) | Classes = {Left,Right,Ambi} | Angular | 1 | [0,6,11;53,0,4] |
| — | — | Frontal | 1 | [2,4,10;54,0,4] |
| — | — | Lateral | 1 | [0,6,5;59,0,4] |
| — | — | Temporal | 1 | [1,5,5;59,0,4] |
| AED (2by3) | Classes = {0,1,2plus} | Angular | 1 | [0,2,0;21,0,0] |
| — | — | Frontal | 1 | [0,2,1;20,0,0] |
| — | — | Lateral | 1 | [0,2,0;21,0,0] |
| — | — | Temporal | 0.68 | [0,2,2;19,0,0] |
| LTGTC (2by3) | Classes = {1-5,6-20,21plus} | Angular | 0.54 | [4,21,2;20,3,8] |
| — | — | Frontal | 0.19 | [1,24,3;19,3,8] |
| — | — | Lateral | 0.79 | [1,24,1;21,1,10] |
| — | — | Temporal | 0.19 | [0,25,2;20,2,9] |
| SG freq (2by3) | Classes = {0,1-2,3plus} | Angular | 1 | [9,60,0;0,0,2] |
| — | — | Frontal | 1 | [11,58,0;0,0,2] |
| — | — | Lateral | 1 | [5,64,0;0,0,2] |
| — | — | Temporal | 1 | [6,63,0;0,0,2] |
| CP freq (2by3) | Classes = {6to10,1to5,0} | Angular | 0.24 | [0,3,5;13,6,45] |
| — | — | Frontal | 0.23 | [2,1,2;16,8,43] |
| — | — | Lateral | 1 | [0,3,1;17,4,47] |
| — | — | Temporal | 0.23 | [1,2,0;18,5,46] |
| tSSS-3cat (2by3) - Broad | Classes = {LowerTertile,MidTertile,UpperTertile} | Angular | 0.39 | [5,15,3;24,3,24] |
| — | — | Frontal | 0.15 | [5,15,1;26,6,21] |
| — | — | Lateral | 0.33 | [2,18,0;27,3,24] |
| — | — | Temporal | 0.15 | [4,16,2;25,0,27] |
| MEGnet-3cat (2by3) - Broad | Classes = {LowerTertile,MidTertile,UpperTertile} | Angular | 0.92 | [3,16,5;25,3,22] |
| — | — | Frontal | 0.92 | [5,14,4;26,3,22] |
| — | — | Lateral | 0.92 | [2,17,1;29,2,23] |
| — | — | Temporal | 0.92 | [2,17,2;28,2,23] |
| tSSS-3cat (2by3) - Beta | Classes = {LowerTertile,MidTertile,UpperTertile} | Angular | 0.55 | [5,18,5;22,1,23] |
| — | — | Frontal | 0.55 | [6,17,4;23,2,22] |
| — | — | Lateral | 0.61 | [1,22,3;24,1,23] |
| — | — | Temporal | 0.57 | [3,20,1;26,2,22] |
| MEGnet-3cat (2by3) - Beta | Classes = {LowerTertile,MidTertile,UpperTertile} | Angular | 0.8 | [2,20,5;20,4,23] |
| — | **—** | **Frontal** | **0.09** | **[2,20,8;17,2,25]** |
| — | — | Lateral | 1 | [1,21,2;23,2,25] |
| — | — | **Temporal** | **0.09** | **[4,18,2;23,0,27]** |
